# Synthesis of a [6]rotaxane with singly threaded γ-cyclodextrins as a single stereoisomer

**DOI:** 10.3762/bjoc.15.177

**Published:** 2019-08-01

**Authors:** Jason Yin Hei Man, Ho Yu Au-Yeung

**Affiliations:** 1 Department of Chemistry, The University of Hong Kong, Pokfulam Road, Hong Kong, P. R. China

**Keywords:** cucurbit[6]uril, cyclodextrin, macrocycles, mechanostereoisomer, rotaxane

## Abstract

A series of hetero [4]-, [5]- and [6]rotaxanes containing both cucurbit[6]uril (CB[6]) and γ-cyclodextrin (γ-CD) as the macrocyclic components have been synthesized via a threading-followed-by-stoppering approach. Due to the orthogonal binding of CB[6] to ammonium and γ-CD to biphenylene/tetra(ethylene glycol), the [*n*]rotaxanes display a specific sequence of the interlocked macrocycles. In addition, despite of the asymmetry of γ-CD with respect to the orthogonal plane of the axle, only one stereoisomer of the [6]rotaxane was obtained.

## Introduction

Cyclodextrins (CDs) are macrocycles composed of glucoses linked via α-1,4-glycosidic bonds. CDs of six (α-CD), seven (β-CD) and eight (γ-CD) glucose units are important molecular hosts that show binding to a wide range of compounds and find applications in different fields including food and pharmaceutical industries [1–5]. CDs are of cone shape with two different openings: a narrower primary face with primary hydroxy groups and a wider secondary face with secondary hydroxy groups [6–7]. With the hydrophobic cavity and the hydrophilic hydroxy groups, CDs efficiently form host–guest complexes with different hydrophobic guests in aqueous medium [8–9]. Among the common CDs, γ-CD possesses a relatively large cavity size that could accommodate up to two aromatic guests to form 1:2 host–guest complexes, in contrast to the usual formation of 1:1 complex by α-CD and β-CD [10–11]. In addition to host–guest chemistry, the favourable binding of CDs to common organic scaffolds has also made CDs popular building blocks for the construction of complex molecular topology such as rotaxanes and catenanes [12–15]. For example, by making use of hydrophobic-driven binding of simple alkyl groups in water to α-CD, Ogino has reported one first example of a rotaxane assembly featuring an alkyldiamine threaded through an α-CD [16]. The corresponding rotaxane with a β-CD was obtained in lower yield, probably due to a weaker binding of the alkyl group to β-CD as a result of a size mismatch [17]. For β-CD with a larger cavity, interlocked molecules derived from aromatic units such as biphenyl, stilbene, cyanine and azobenzene have also been reported [18–24]. High order oligorotaxanes with a multiple number of α- or β-CDs threaded through polymer chains have also been prepared [25–28]. The interlocked CDs have been shown to provide an interesting insulating effect to π-conjugated polymers [29–31].

On the other hand, γ-CD is relatively less employed in the synthesis of mechanically interlocked molecules despite of its ability to form interesting 1:2 inclusion complexes, and there are only few examples of rotaxane and catenane featuring γ-CD as an interlocked macrocycle [32–36]. By adopting a stepwise stoppering approach, Anderson and co-workers have synthesized a [3]rotaxane consisting of two different axles, derived from a stilbene and a cyanine, threaded through one γ-CD [33]. Inouye has also reported a [3]rotaxane with two pyrene-derived axles threaded through one γ-CD [34]. More recently, Yang and co-workers have described a rotaxane-based host that detects tryptophan which binds to the γ-CD cavity of the rotaxane [35]. As part of our program in the synthesis and application of complex, multicomponent interlocked molecules [37–39], we are interested in exploiting the binding capability of γ-CD in the construction of high order [*n*]rotaxanes and [*n*]catenanes. Here, we report our work on the synthesis of multiring, hetero[*n*]rotaxane containing γ-CD and cucurbit[6]uril (CB[6]) as the macrocyclic components in an aqueous medium. Because of the orthogonal binding of γ-CD to biphenylene and tetra(ethylene glycol) and CB[6] to ammonium, [*n*]rotaxanes of only a specific sequence of the interlocked macrocycles were obtained despite of the possibility of other sequence isomers. In addition, the three γ-CDs in the [6]rotaxane were found to adopt only one orientation, possibly due to inter-ring interactions with the CB[6], to give the [6]rotaxane as one single stereoisomer. Considering the ability of γ-CD to form stable 1:2 inclusion complexes, these singly threaded [*n*]rotaxanes could serve as an entry point to other high order interlocked structures by further interlocking at the γ-CD.

## Results and Discussion

### Building block design and rotaxane synthesis

**Scheme 1:**
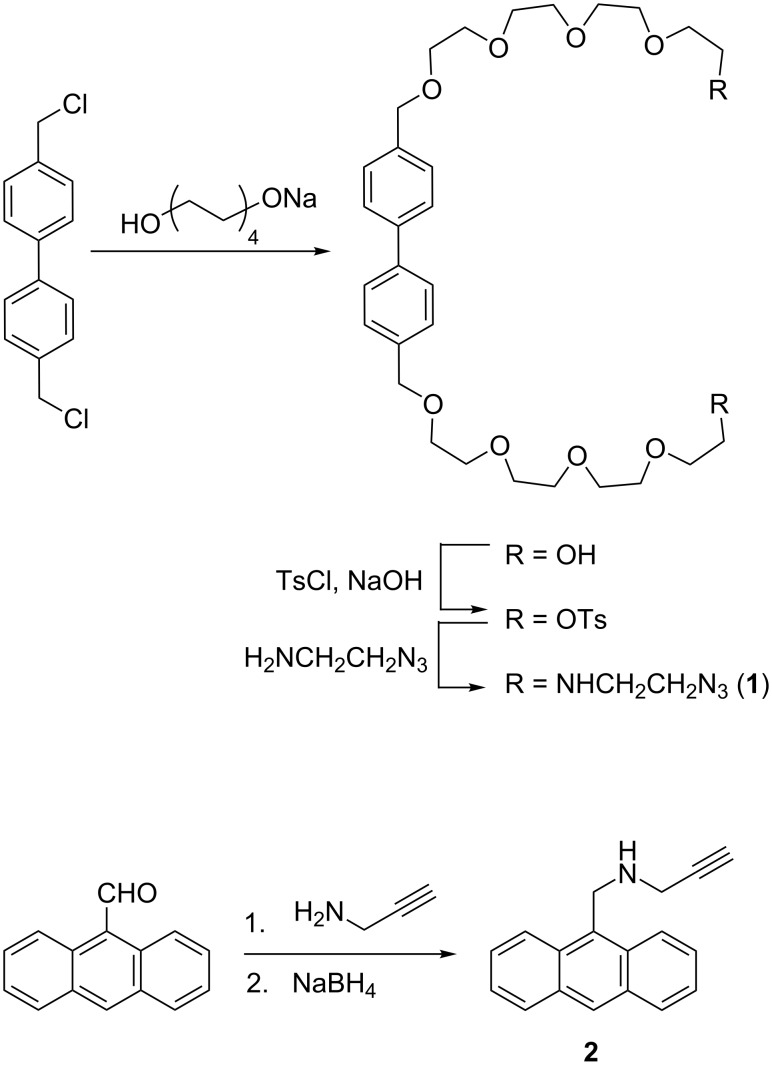
Synthesis of the axle and stopper building blocks **1** and **2**.

To encourage complex formation with γ-CD, axle **1** was designed with a biphenylene core to bind to the macrocycle through hydrophobic effect. The axle is terminated by 2-aminoethyl azide for CB[6]-mediated azide–alkyne cycloaddtion (CBAAC) with the anthracene-derived propagylamine stopper **2**. In CBAAC, the strong ammonium–CB[6] binding places the alkyne and azide in a close proximity inside the CB[6] cavity and facilitates the cycloaddition [40–42]. Since CB[6] binding is required for the covalent bond formation, interlocking of the macrocycle is ensured to result in a good efficiency of mechanical bond formation. The synthesis of **1** and **2** is depicted in Scheme 1.

**Scheme 2:**
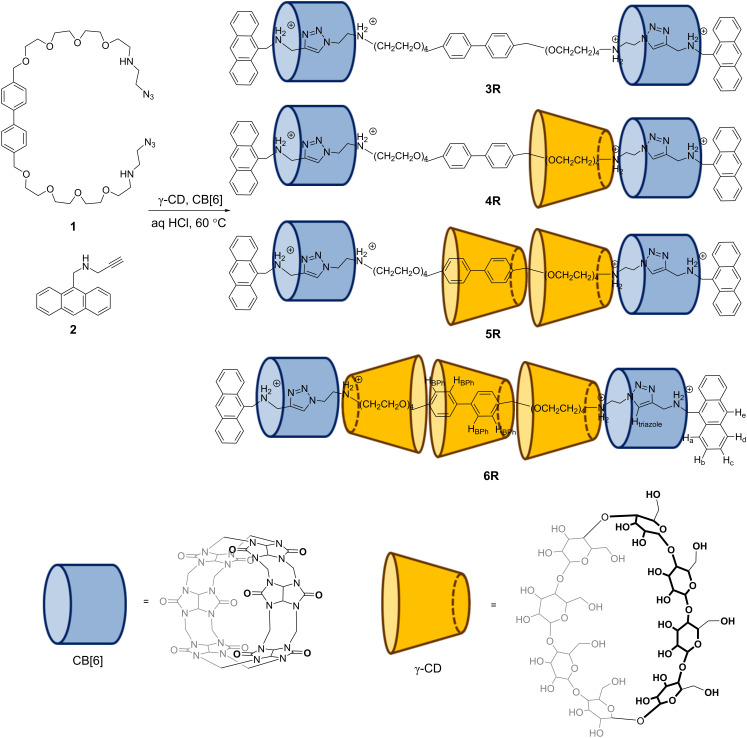
Synthesis of [*n*]rotaxanes **3R** to **6R**. Note that the position and orientation of the γ-CD are arbitrary.

**Figure 1:**
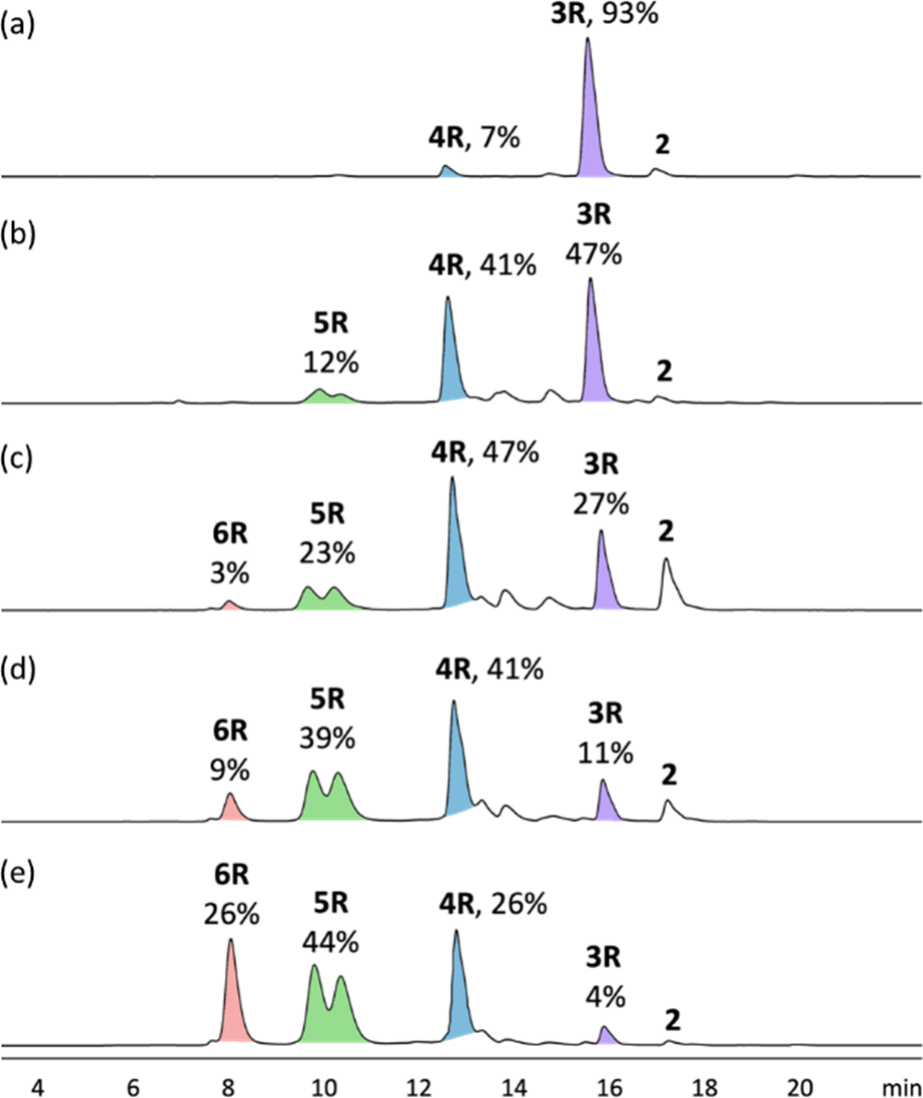
LC–MS analysis of the reaction mixture of the [*n*]rotaxane synthesis in the presence of (a) 0.5; (b) 5; (c) 10; (d) 20; and (e) 50 equiv of γ-CD.

To synthesize hetero[*n*]rotaxanes containing both γ-CD and CB[6], a 1:1 mixture of the anthracene stopper **2** and CB[6] in 50 mM HCl was first heated at 100 °C for 5 minutes in a microwave reactor to facilitate the dissolution of the CB[6] (Scheme 2). The solution was then added to a solution mixture of the biphenylene building block **1** and γ-CD at different ratios, and the mixture was heated at 60 °C overnight. The products were analyzed by LC–MS. Contrary to most reports on the inclusion of simple aromatic or poly(ethylene glycol) in γ-CD where a 2:1 binding stoichiometry was observed, LC–MS analysis of the reaction mixture containing a 2:1 molar ratio of **1**/γ-CD stoppered by **2** showed only the [3]rotaxane **3R** (93%, *m*/*z* = 798.0, 4+; 1063.8, 3+) and a small amount of the [4]rotaxane **4R** (7%, *m*/*z* = 1122.0, 4+) with no doubly threaded product observed. The low efficiency of γ-CD interlocking may be due to the weak γ-CD binding under the reaction conditions, especially when heat was applied for the CBAAC. Electrostatic repulsion between the positively charged ammoniums under the acidic reaction conditions may also weaken the formation of the 2:1 complex. To promote γ-CD inclusion in the final rotaxane product, a larger amount of γ-CD was used. When 5 equiv of γ-CD was used, the yield of **4R** increases significantly to 41% at the expense of **3R**. Interestingly, a small amount of the [5]rotaxane **5R** (12%, *m*/*z* = 1446.7; 4+) was also observed. Further increasing the amount of γ-CD gave more of the higher order **5R** and even the [6]rotaxane **6R** (*m*/*z* = 1416.7, 6+; 1770.6, 4+), with the latter obtained in 26% yield when 50 equiv of γ-CD was used (Figure 1). Of note, while **3R**, **4R** and **6R** appear as a single peak in the LC chromatogram, two closely eluted peaks with mass signals consistent with the [5]rotaxane were observed for **5R**, suggesting the existence of two or more isomeric forms of **5R** which are non-interconvertible on the chromatographic time scale. The [*n*]rotaxanes were all purified by preparative HPLC and characterized by ^1^H NMR, ^13^C{^1^H} NMR, HRESIMS, and tandem MS. The HRESIMS spectra of **3R**, **4R**, **5R** and **6R** all show an isotopic pattern that is consistent with the respective molecular formula (Figures S20–S23 in Supporting Information File 1). In the MS/MS spectra, fragments corresponding to the loss of the γ-CD and the anthracene stoppers were observed, suggesting the breaking of the glycosidic bonds of the γ-CD and the anthracene–CH_2_(NRH_2_)^+^ bond as the major fragmentation pathways (Figures S24–S27 in Supporting Information File 1).

### NMR structures of the hetero[*n*]rotaxanes

**Figure 2:**
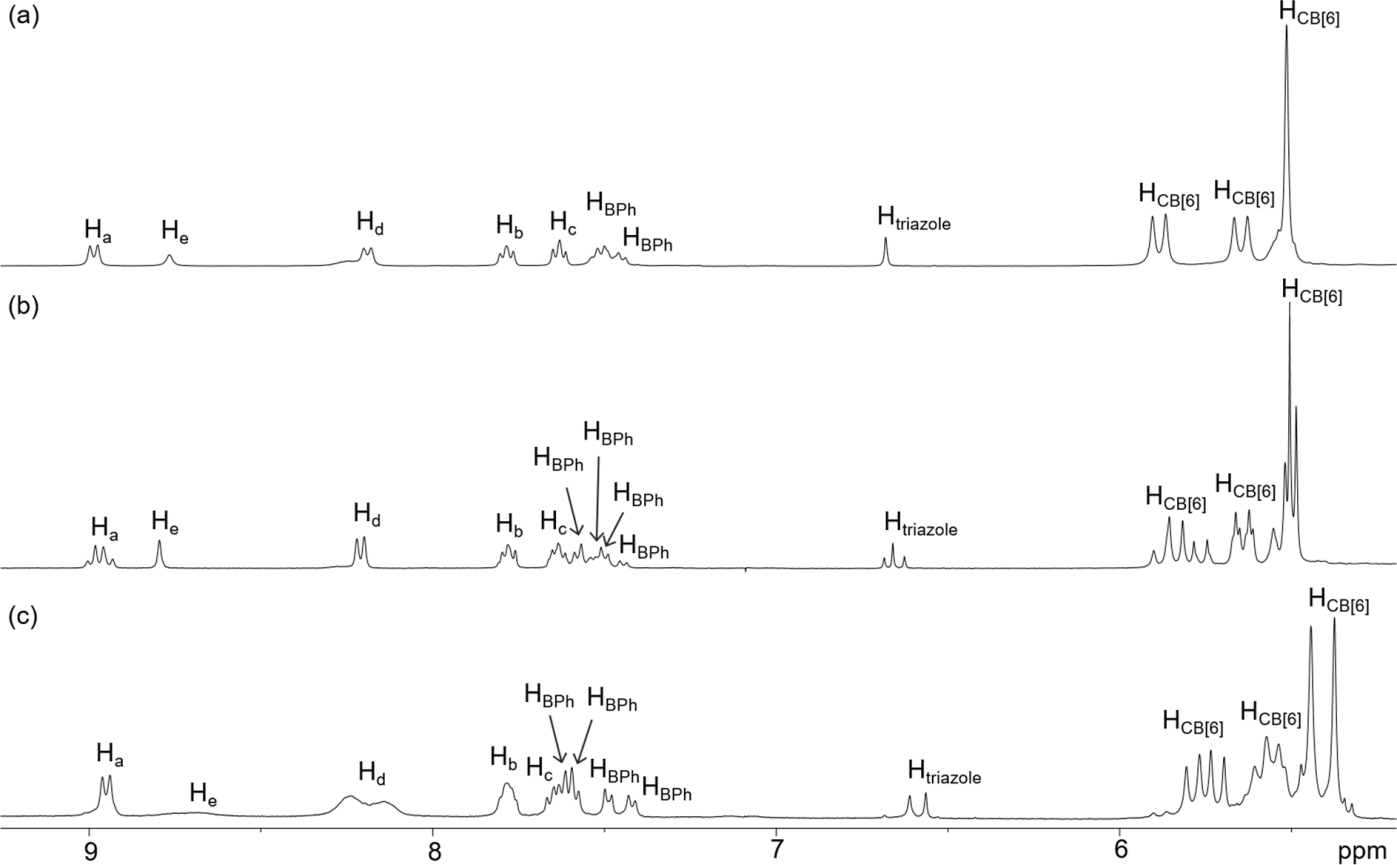
Partial ^1^H NMR spectra (500 MHz, D_2_O, 298 K) of (a) **6R**; (b) **5R**; and (c) **4R**.

Structures of the hetero[*n*]rotaxanes were further characterized by ^1^H NMR in D_2_O. Interestingly, the structurally most complex **6R** seems to be the most symmetrical one among the three hetero[*n*]rotaxanes, our discussion on the NMR therefore begins with **6R**. The ^1^H spectrum of **6R** contains one set of sharp signals, suggesting that the [6]rotaxane is adopting a relatively rigid and symmetrical structure in solution (Figure 2a). NOE cross peaks between the triazole and CB[6] protons, and also H_a_ of the anthracene stoppers and CB[6] protons were observed, suggesting that the two CB[6] are interlocking at the triazole which is consistent with the strong binding of the CB[6] to the ammoniums (Figure S18 in Supporting Information File 1). Due to the severe peak overlapping in the aliphatic region at 3.5–4.5 ppm, NOE cross peaks in that regions cannot be unambiguously assigned and the relative position of the γ-CD on the axle cannot be further probed without uncertainty. Nevertheless, with the other three interlocked γ-CD, the biphenylene and the two tetra(ethylene glycol) parts should all be threaded inside the γ-CD.

**Figure 3:**
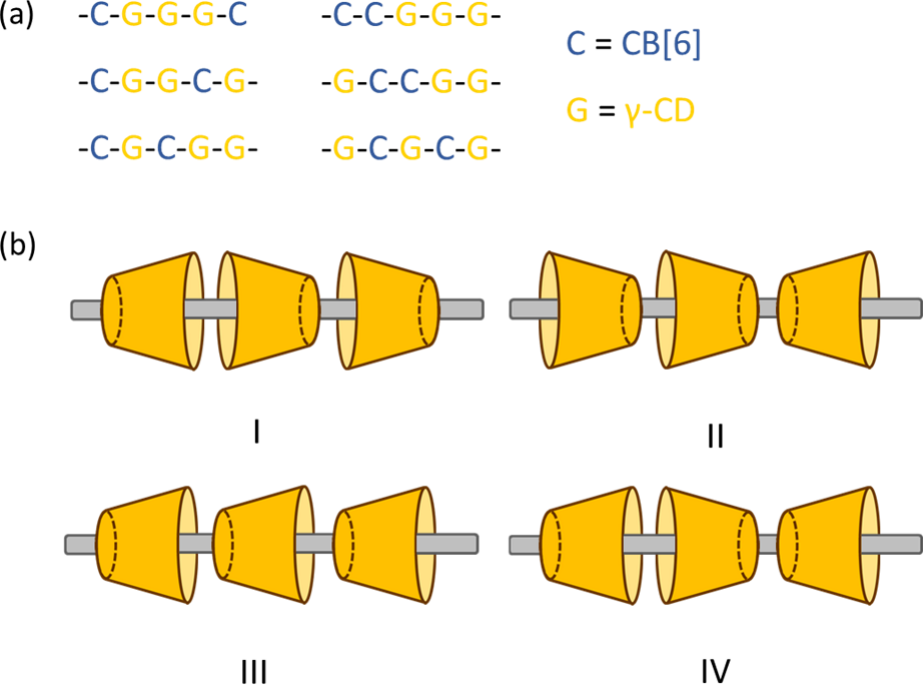
(a) All the possible sequences of a [6]rotaxane with two CB[6] and three γ-CD interlocked on an axle. Only the sequence on the top left was observed in **6R** (with the orientation of the γ-CD disregarded). (b) The four possible relative orientations of the three γ-CD in **6R**.

The overall structure of **6R** therefore features a specific CB[6]–(γ-CD)_3_–CB[6] sequence of the interlocked macrocycles. Of note, for a [6]rotaxane with two types of five interlocked macrocycles on one axle, there could be in total six different possible sequences (Figure 3a). The emergence of only one particular sequence here is a result of the specific and orthogonal interactions of the macrocycles with different part of the axle. More interestingly, only one set of signals was observed for both the triazole and CB[6] protons in **6R**. This observation is suggesting that 1) **6R** is adopting a structure that the CB[6] at the termini are far from the influence of the asymmetry of the γ-CD in the center; and 2) the two γ-CD next to the CB[6] are pointing to the CB[6] with the same face, resulting in a symmetry plane orthogonal to the axle when the central γ-CD is disregarded. Considering that the primary and secondary faces of a γ-CD are different, there could be four possible orientations of the three interlocked γ-CD to give four different triazole environments (Figure 3b). Yet, only one of the four (isomer I or II) possible isomers has been observed in the ^1^H NMR, suggesting that **6R** was obtained as a single stereoisomer. In fact, cooperative binding between CB[6] and cyclodextrins via hydrogen bonds in rigid (pseudo)rotaxane systems has been previously reported [43–48]. The full occupancy of the axle by five macrocycles and the resulting close proximity of the macrocycles in **6R** may hence resulted in similar interactions that stabilize a particular stereoisomer over the others, thus making the **6R** synthesis stereoselective. Moreover, as the secondary face in γ-CD (diameter ≈ 8.3 Å [49]) is much wider than the rim of CB[6] (diameter ≈ 5.8 Å [50]), it is expected that the primary face of γ-CD (diameter ≈ 7.5 Å [49]) will have a better size match and hence a stronger interaction with the CB[6], and that the two γ-CD adjacent to the CB[6] may be oriented with the primary face towards the anthracene terminal [43]. Of note, it has been reported that for the interactions between the smaller α-CD and CB[6], it is the wider secondary face of α-CD (diameter ≈ 5.2 Å; primary face diameter ≈ 4.7 Å [49]) being complementary in size and showed preferred interactions with CB[6] [45]. Nevertheless, more detailed binding studies and structural characterization will be required for a definitive assignment of the γ-CD orientations and elucidating the origin of the stereoselectivity in the synthesis of **6R**.

**Figure 4:**
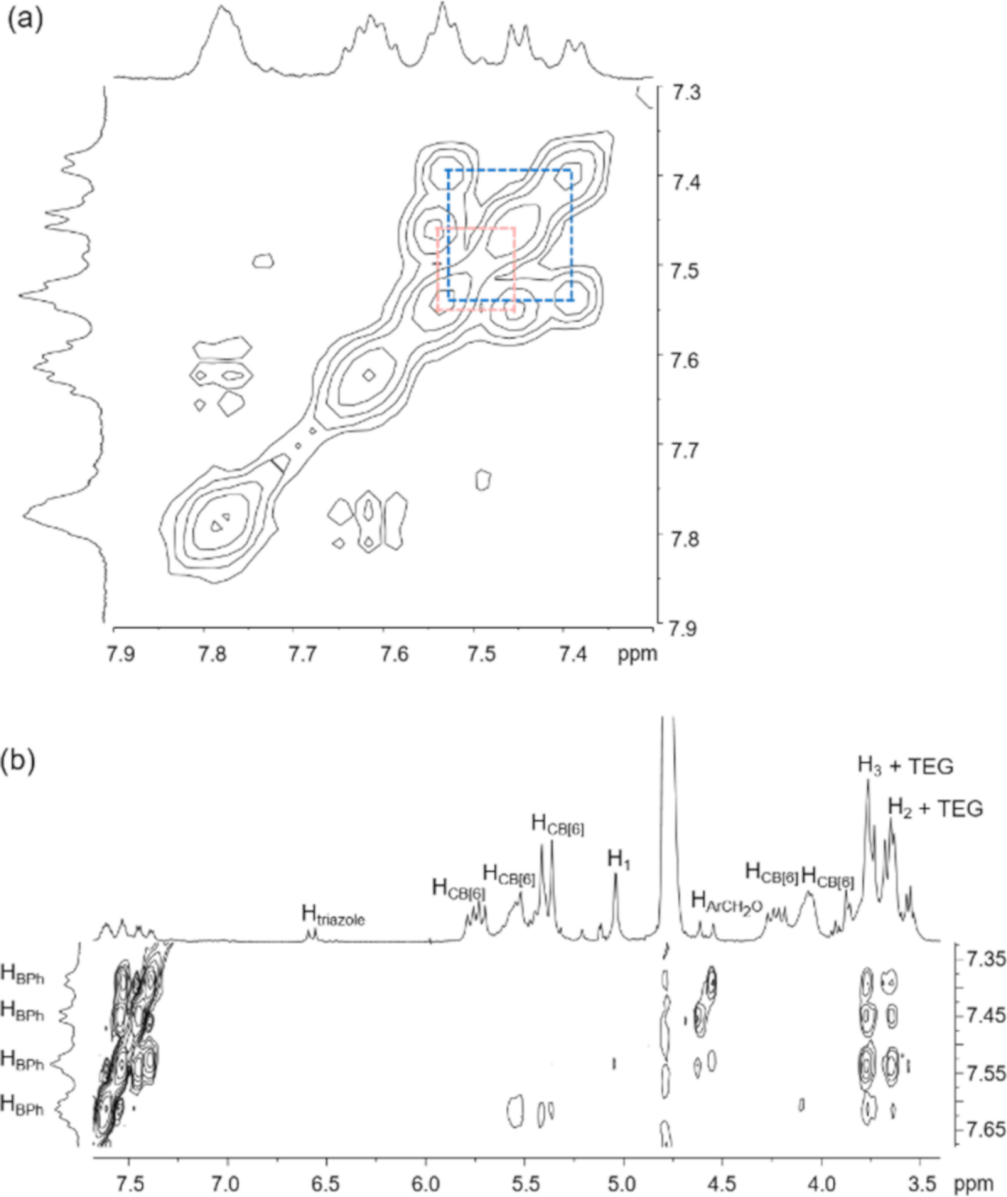
Partial (a) COSY spectrum (500 MHz, D_2_O, 298 K) and (b) NOESY (500 MHz, D_2_O, 298 K) of **4R** showing the H_BPh_ protons respectively as two paired of coupled signals and their NOE cross peaks with the H_2_ and H_3_ protons.

The ^1^H spectrum of **4R** is generally similar to that of **6R** and strong NOE cross peaks between the triazole and CB[6], and also H_a_ of the anthracene and CB[6] protons were observed (Figure S10 in Supporting Information File 1), confirming that the two CB[6] are stationing at the ammonium/triazole positions similar to that in **6R**. Yet, there are few significant differences in the spectrum of **4R** noted: 1) two singlets were observed for the triazole; 2) the CB[6] was observed as two different sets of signals; and 3) two pairs of coupled signals and two singlets were observed for the biphenylene and ArC*H*_2_O protons (Figure 2b, Figure 4a and Supporting Information File 1, Figure S7). These observations suggest that the two termini of **4R** are more different than that of **6R**, which should be originated from the asymmetry of the interlocked γ-CD. Although the hydrophobic biphenylene is expected to form a more stable inclusion complex with γ-CD due to a stronger hydrophobic effect, a structure with the γ-CD interlocking at the central biphenylene of **4R** may not explain the non-equivalent chemical environments of the two termini. As discussed in the structure of **6R** above, the central γ-CD at the biphenylene position has little influence on the symmetry of the triazole and CB[6] at the end of the rotaxane. Together with the relatively different chemical environments for the two ends of the biphenylene, the γ-CD in **4R** is therefore proposed to be interlocking at one of the tetra(ethylene glycol) moieties, possibly as a result of the stabilizing interactions between the γ-CD and CB[6]. Indeed, due to the strong interactions between CB[6] and cyclodextrin, it has been reported that CB[6] can dissemble a [3]pseudorotaxane consisting of two linear axles bound in the cavity of a γ-CD to form a [4]pseudorotaxane consisting of one γ-CD and two CB[6], and that because of the steric hindrance provided by the CB[6], inclusion of a second guest in the γ-CD cavity is discouraged [46]. The observation that no rotaxane product derived from a 2:1 complex of **1**/γ-CD was obtained could also be attributed to a similar steric effect if the CB[6] and γ-CD are in close proximity, which is also consistent with the proposed structure of **4R**.

Again, the γ-CD is proposed to face the CB[6] with its primary face due to the better size match and stronger interactions. In addition, NOE cross peaks between the biphenylene protons and two signals at ca. 3.6–3.8 ppm were observed (Figure 4b). Although the resonances of H_2_ and H_3_ are overlapped with that of the tetra(ethylene glycol) at 3.6–3.8 ppm, no NOE cross peak between the biphenylene and tetra(ethylene glycol) was observed in the most flexible **3R** (Figure S6 in Supporting Information File 1), suggesting that the observed NOE in **4R** are likely due to a close proximity of the central biphenylene with the secondary face of the γ-CD, further suggesting the γ-CD is interacting with the CB[6] via its narrower primary face. In previous reports of (pseudo)rotaxane systems when a cooperative cyclodextrin and CB[6] binding was observed, an NOE correlation between the cyclodextrins and CB[6] protons could be observed due to their close proximity [45–46]. In our case, due to the severe overlapping of the signals in the aliphatic region, no clear correlation between the γ-CD and CB[6] could be identified in **4R**. Despite of the possibility that the γ-CD could move along the axle and shuttle between the two tetra(ethylene glycol) parts via the biphenylene, this proposal is not considered as a fast moving γ-CD along the axle on the NMR timescale will probably average out the chemical environment of the central part of the axle and should not give the distinct aromatic and ArC*H*_2_O signals for the biphenylene. Indeed, the ^1^H NMR spectrum of **4R** obtained at 338 K showed a significant sharpening of H_d_ and H_e_, and the chemical shifts of the biphenylene aromatic and methylene and CB[6] protons are less resolved when compared to those resonances observed at room temperature, showing probably a faster γ-CD dynamic at 338 K that averaged out the chemical environments of these protons (Figure S19 in Supporting Information File 1).

**Figure 5:**
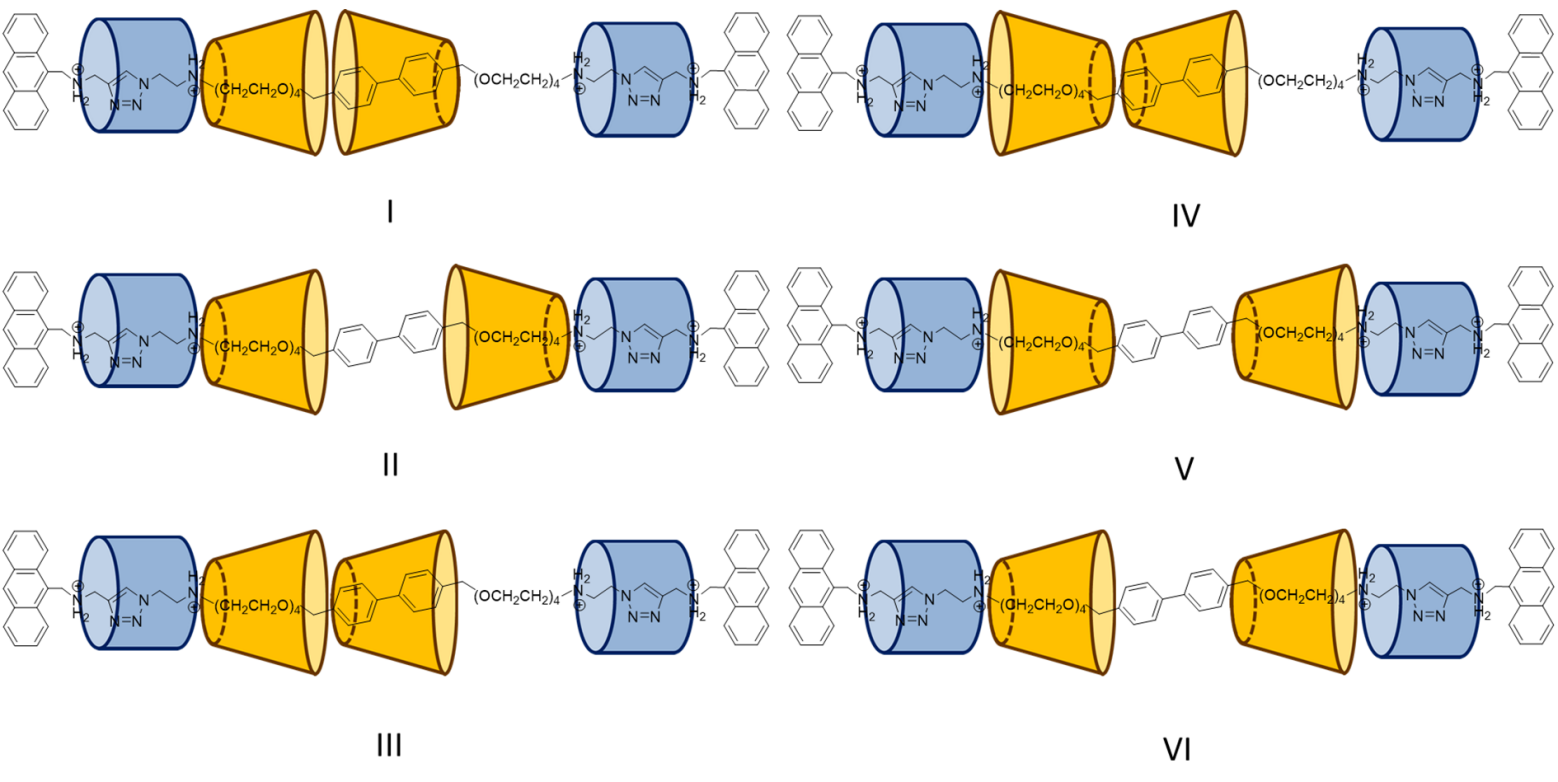
Possible structures of **5R** assuming no fast shuttling of the γ-CD along the axle.

The ^1^H spectrum of **5R** is much more complex. H_a_ of the anthracene stopper was observed as two overlapping doublets, and three triazole singlets and more than two sets of CB[6] signals were observed, showing that **5R** was obtained as a mixture of stereoisomers with very different chemical environments at the termini. The existence of a stereoisomeric mixture of **5R** is also consistent with the two closely eluted peaks in the LC chromatogram. Assuming no fast movement of the two γ-CDs on the axle in **5R** as in **4R**, there could be six different possible isomers for **5R**, and if any γ-CD interlocking at the tetra(ethylene glycol) part is interacting with the CB[6] via its primary face just as in **4R** and **6R**, three of the six possible isomers (IV, V and VI) will be eliminated (Figure 5). If it is further assumed that the γ-CD on the biphenylene has no influence on the symmetry of the triazole and CB[6] as discussed above, isomers I and III will both give two sets, whereas the symmetrical isomer II will give one set of triazole and CB[6] signals. The NOESY spectrum of **5R** may not provide further information due to the complexity of the spectrum (Figure S14 in Supporting Information File 1), and further structural assignment of **5R** may not be straightforward unless more detailed structural characterization such as X-ray crystal data is available. Nevertheless, the observation that **5R** was obtained as different stereoisomers further strengthens the proposal that it is the full occupancy of the axle by the macrocycles in **6R** that results in a cooperative interaction so that the formation of **6R** is stereoselective. With one less γ-CD in **5R**, interactions between the axle and the macrocycles alone are only enough for interlocking the macrocycle, but not sufficient to drive a specific orientation of all the γ-CD, suggesting intercomponent stabilization could play an important role in directing higher level structures in mechanically interlocked molecules, reminiscent to the intra-strands interactions that stabilize high-order structures in (bio)polymers.

## Conclusion

In summary, complex hetero[n]rotaxanes have been efficiently obtained by using both CB[6] and γ-CD which are interlocked onto the axle by orthogonal interactions. Of note, the γ-CD in all the rotaxanes are only singly threaded, suggesting that further guest molecules could bind to the γ-CD cavity to give interesting host–guest complexes with the [*n*]rotaxanes as the host, or these [*n*]rotaxanes being further developed into more complex interlocked structures. Despite of the different possible sequences of the interlocked macrocycles, these hetero[*n*]rotaxanes were obtained with only a specific sequence with the two CB[6] located at the end. Moreover, the synthesis of **6R** is stereoselective and only one stereoisomer was formed, probably due to the cooperative interactions between the CB[6] and the γ-CD on the fully occupied axle of **6R**. These findings will provide better insight on the use of intercomponent interactions to control the stereochemistry of complex, multicomponent rotaxane, catenane and other mechanically interlocked architectures and the potential applications thereof.

## Supporting Information

File 1 Detailed experimental procedures of the syntheses and characterization data (MS, MS^2^, ^1^H and ^13^C NMR spectra).
