# Supplementary material for: Synthesis of a [6]rotaxane with singly threaded γ-cyclodextrins as a single stereoisomer
Source: Beilstein J Org Chem. 2019 Aug 1;15:1829–37. doi: 10.3762/bjoc.15.177 (PMC6693375; doi:10.3762/bjoc.15.177)
Supplement: File 1 — Detailed experimental procedures of the syntheses and characterization data (MS, MS2, 1H and 13C NMR spectra). [file Beilstein_J_Org_Chem-15-1829-s001.pdf]

BEILSTEIN JOURNAL OF ORGANIC CHEMISTRY

Supporting Information

for

Synthesis of a [6]rotaxane with singly threaded γ-cyclodextrins as a single stereoisomer

Jason Yin Hei Man and Ho Yu Au-Yeung

Beilstein J. Org. Chem. **2019,** *15,* 1829–1837. doi:10.3762/bjoc.15.177

Detailed experimental procedures of the syntheses and characterization data (MS, MS^2, ^1H and ^{13}C NMR spectra)

License and Terms: This is a supporting information file under the terms of the Creative Commons Attribution License (http://creativecommons.org/licenses/by/4.0). Please note that the reuse, redistribution and reproduction in particular requires that the authors and source are credited.
The license is subject to the *Beilstein Journal of Organic Chemistry* terms and conditions: (https://www.beilstein-journals.org/bjoc)

Table of contents

1.	*Synthesis*	S2
2.	*NMR*	S6
3.	*ESIMS*	S20
4.	*References*	S27

1. Synthesis

General. All reagents were purchased from commercial suppliers (Aldrich, Dkmchem and J & K) and used without further purification. All solvents for syntheses were of analytical grade (ACI Labscan and DUKSAN Pure Chemicals). MeCN, $CHCl_3$ and MeOH were distilled over CaH_2 before use. Compound **1**, 2-azidoethylamine and cucurbit[6]uril (CB[6]) were synthesized according to literature procedures.[1,2] Microwave-assisted reactions were carried out using a Discover SP microwave synthesizer (CEM, USA) in the closed vessel focused single. Thin layer chromatography (TLC) was performed on silica gel 60 F254 (Merck, Germany, aluminium sheet) and column chromatography was carried out on silica gel 60F (Silicycle, Canada). HPLC analyses were carried out using a Waters-Alliance e2695 system coupled to a 2489 UV–vis detector. ESIMS analyses were carried out using a Waters-Acquity UPLC H-Class system coupled with a QDa MS detector. NMR spectra were recorded on Bruker DPX spectrometers with working frequencies of 400 MHz or 500 MHz for ^1H, and 100 MHz or 125 MHz for ^{13}C, respectively. Chemical shifts are reported in ppm and referenced to residual solvent signals (for ^1H: $CDCl_3$: δ = 7.26 ppm, D_2O: δ = 4.79 ppm; For ^{13}C: $CDCl_3$: δ = 77.16 ppm).

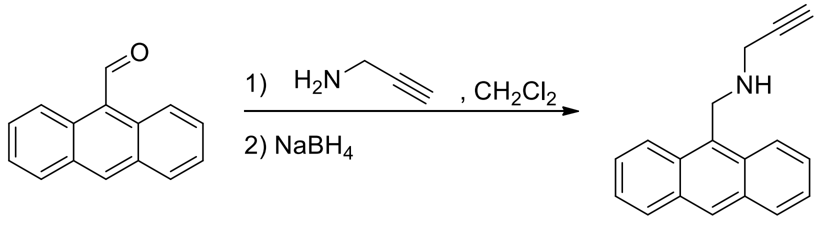

2 , 55%

Scheme S1. Synthesis of building block **2**.

Synthesis of **2**. A mixture of 9-anthracenecarboxyaldehyde (0.40 g, 1.94 mmol) and propargylamine (0.12 g, 2.13 mmol) in dry CH_2Cl_2 (20 mL) was stirred at room temperature overnight. The reaction mixture was cooled to 0 °C and $NaBH_4$ (80 mg, 1.94

mmol) was added in portions, followed by the addition of dry MeOH (5 mL). The reaction mixture was stirred at room temperature for 2 h. The solvents were removed by a rotary evaporator and the residue was dissolved in CH_2Cl_2 (30 mL). The organic solution was washed with water (2 × 10 mL), brine, dried over $MgSO_4$, concentrated and purified by column chromatography on silica (CH_2Cl_2). A pale yellow solid was obtained. Yield = 0.26 g, 55%. ^{1}H NMR (500 MHz, $CDCl_3$, 298 K) δ = 8.43 (d, *J* = 9.2 Hz, 2H), 8.40 (s, 1H), 8.01 (d, *J* = 4.2 Hz, 2H), 7.57-7.54 (m, 2H), 7.49-7.46 (m, 2H), 4.89 (s, 2H), 3.65 (d, *J* = 2.4 Hz, 2H), 2.45 (t, *J* = 2Hz, 1H). ^{13}C{^{1}H} NMR (125 MHz, $CHCl_3$, 298K) δ = 131.6, 130.7, 130.6, 128.24, 127.6, 126.3, 125.1, 124.2, 82.6, 72.1, 44.4, 38.5.

General synthesis of the rotaxanes. A solution of **2** (10 mg, 0.042 mmol) and CB[6] (42 mg, 0.042 mmol) in 0.05 M HCl (1.4 mL) was heated at 100 °C for 5 min in a microwave reactor. The solution was then added to a mixture of building block **1** (10 mg, 0.11 mmol) and γ-cyclodextrin in 0.05 M HCl (1.4 mL) over 1 h and the reaction was heated at 60 ^{o}C for overnight. The rotaxanes were purified by preparative HPLC using a Waters-Alliance e2695 system coupled to a 2489 UV–vis detector using a C18 SunFire preparative columns (5 μm, 10 × 250 mm or 10 μm, 4.6 × 250 mm) with a gradient elution described below at a flow rate of 3 μL/min. UV–vis absorbance was monitored at 247 nm.

Elution gradient:

time/min	H_2O (with 0.1% TFA)	MeCN (with 0.1% TFA)
0	75%	25%
3	75%	25%
6	70%	30%
7	70%	30%
16	55%	45%
18	40%	60%
19	0%	100%
22	0%	100%

3R. ^{1}H NMR (400 MHz, D_2O, 298 K) δ = 8.93 (d, *J* = 9.1 Hz, 4H), 8.17 (s, 2H), 7.85 (t, *J* = 7.6 Hz, 4H), 7.76 (d, *J* = 8.0 Hz, 4H), 7.68 (br, 4H), 7.62 (t, 5.8 Hz, 4H), 7.51 (d, *J* = 8.0

Hz, 4H), 6.43 (s, 2H), 5.60 (d, 15.5 Hz, 12H), 5.47 (s, 4H), 5.31 (d, *J* = 15.4 Hz, 12H), 5.16 (s, 24H), 4.60 (s, 8H), 4.07 (d, *J* = 15.5 Hz, 12H), 4.00–3.96 (m, 8H), 3.84–3.78 (m, 20H), 3.74–3.69 (m, 16H), 3.59–3.56 (m, 8H). $^{13}C\{^1H\}$ NMR (100 MHz, D_2O, 298 K) δ = 163.1, 162.8, 156.0, 155.9, 146.3, 143.7, 139.0, 138.9, 137.4, 131.6, 131.4, 131.0, 130.9, 129.6, 129.3, 127.2, 126.6, 125.9, 125.7, 125.3, 125.0, 123.7, 122.1, 120.0, 119.9, 118.0, 115.1, 110.1, 72.6, 70.0, 69.9, 69.4, 65.9, 51.4, 51.0, 47.3, 45.9, 45.6, 45.1, 44.5. ESI-MS: 798.0 $[M+4H]^{4+}$; 1063.5 $[M+3H]^{3+}$.

4R. ^1H NMR (400 MHz, D_2O, 298K) δ = 8.95 (d, *J* = 9 Hz, 4H), 8.24 (br, 4H), 8.13 (br, 2H), 7.78 (br, 4H), 7.67–7.78 (m, 8H), 7.50–7.41 (m. 2H), 6.59 (d, *J* = 19 Hz, 2H), 5.25 (q, *J* = 13 Hz, 12H), 5.61–5.52 (m, 12H), 5.44–5.40 (m, 24H), 5.12–5.03 (m, 8H), 4.63 (s, 2H), 4.68 (s, 2H), 4.57 (s, 2H), 4.29–4.18 (m, 12H), 4.12–4.04 (m, 12H), 3.87 (s, 4H), 3.81–3.73 (m, 20H), 3.69–3.62 (m, 18H), 3.60–3.51 (m, 6H). $^{13}C\{^1H\}$ NMR (125 MHz, D_2O, 298 K) δ = 163.2, 162.9, 156.4, 156.2, 139.2, 131.3, 131.2, 129.1, 129.1, 127.0, 126.8, 125.8, 120.4, 120.0, 117.6, 115.3, 102.4, 101.7, 81.2, 80.5, 73.1, 73.0, 72.0, 71.8, 70.2, 70.0, 69.9, 69.8, 60.3, 60.0, 51.5, 51.2, 45.6, 45.1. ESI-MS: 1122.0 $[M+4H]^{4+}$.

5R. ^1H NMR (400 MHz, D_2O, 298 K) δ = 8.95 (m, 4H), 8.80 (s, 2H), 8.21 (d, 4H), 7.78 (t, *J* = 8.3 Hz, 4H), 7.64 (t, *J* = 8.4 Hz, 4H), 7.69–7.44 (m, 8H), 6.69–6.63 (m, 2H), 5.90–5.75 (m, 12H), 5.90–5.75 (m, 12H), 5.55 (s, 4H), 5.52–5.49 (m, 24H), 5.12–5.07 (m, 16H), 4.37–4.25 (m, 12H), 4.20 (d, *J* = 19.3 Hz, 12H), 3.96–3.88 (m, 20H), 3.86–3.81 (m, 30H), 3.79–3.75 (m, 22H), 3.73–3.65 (m, 48H), 3.62–3.56 (m, 20H). $^{13}C\{^1H\}$ NMR (125 MHz, D_2O, 298 K) δ = 168.1, 156.5, 156.3, 131.3, 131.2, 129.2, 127.0, 126.9, 125.8, 125.0, 123.0, 109.7, 102.9, 102.6, 102.1, 101.8, 82.1, 81.5, 80.8, 80.6, 73.1, 73.0, 72.4, 72.2, 72.1, 72.0, 71.9, 70.3, 70.3, 70.1, 70.0, 69.9, 69.8, 60.3, 60.2, 51.7, 51.7, 51.6, 51.4, 51.3. ESI-MS: 1446.7 $[M+4H]^{4+}$.

6R. ^1H NMR (400 MHz, D_2O, 298 K) δ = 8.99 (d, *J* = 9.0 Hz, 4H), 8.77 (s, 2H), 8.19 (d, *J* = 8.6 Hz, 4H), 7.79 (t, *J* = 7.8, 4H), 7.63 (t, *J* = 7.8 Hz, 4H), 7.52–7.44 (m, 8H), 6.68 (s, 2H), 5.89 (d, *J* = 15.4 Hz, 12H), 5.65 (d, *J* = 15.4 Hz, 12H), 5.51–5.49 (m, 28H), 5.12–

5.06 (m, 24H), 4.35 (d, J = 15.4 Hz, 12H), 4.19 (d, J = 15.2 Hz, 12H), 3.92–3.86 (m, 48H), 3.83–3.73 (m, 90H), 3.65–3.58 (m, 50H). $^{13}C\{^{1}H\}$ NMR (125 MHz, D_2O, 298 K) δ = 156.5, 156.1, 139.2, 131.3, 131.3, 129.2, 128.8, 127.0, 125.8, 125.0, 122.2, 121.0, 102.9, 102.1, 101.7, 82.1, 80.8, 80.5, 73.1, 73.1, 73.1, 73.0, 72.4, 72.1, 71.9, 71.8, 70.2, 70.0, 69.8, 60.2, 60.2, 60.0, 51.7, 51.4. ESI-MS: 1416.7 $[M+5H]^{5+}$, 1770.6 $[M+4H]^{4+}$.

2. NMR

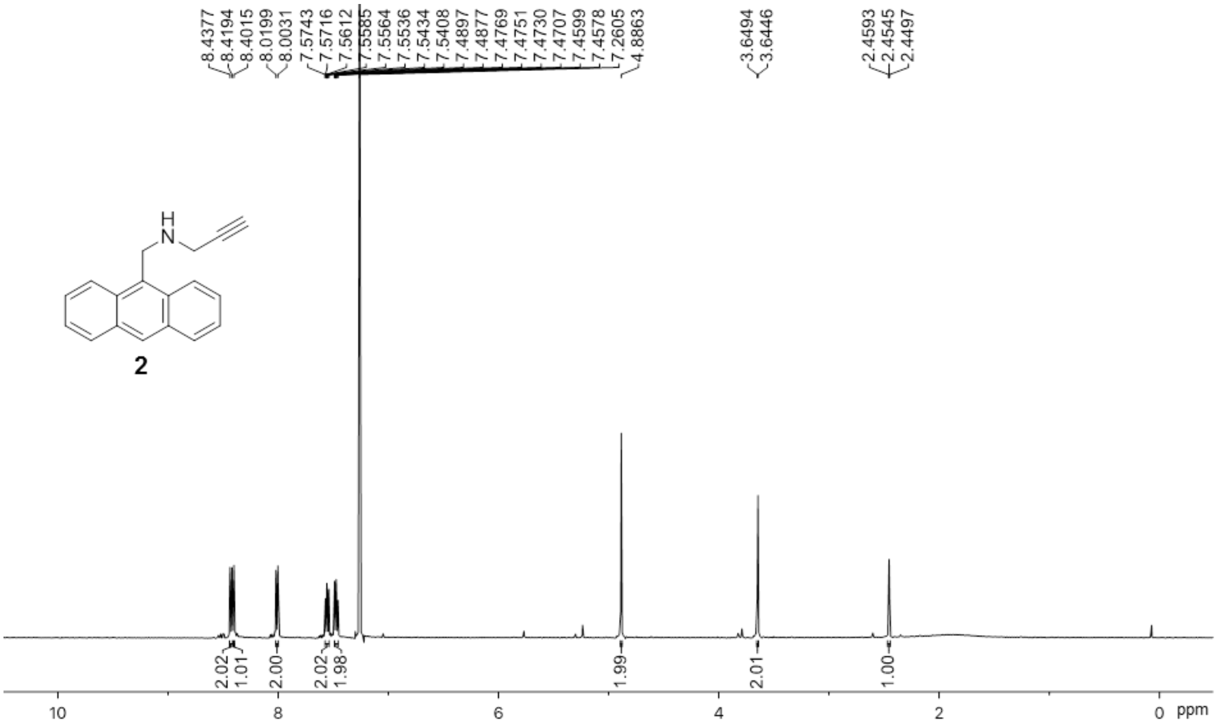

Figure S1. ^{1}H NMR (500 MHz, $CDCl_3$, 298 K) of **2.**

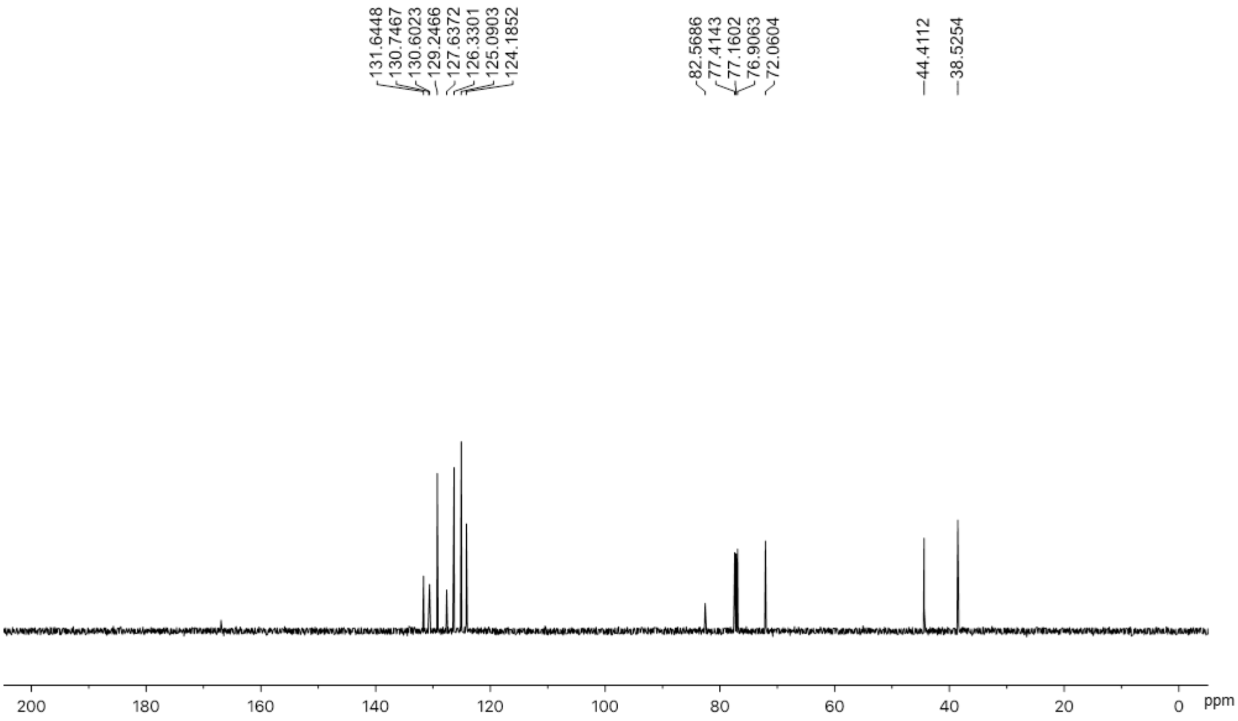

Figure S2. $^{13}C\{^{1}H\}$ NMR (125 MHz, $CDCl_3$, 298 K) of **2.**

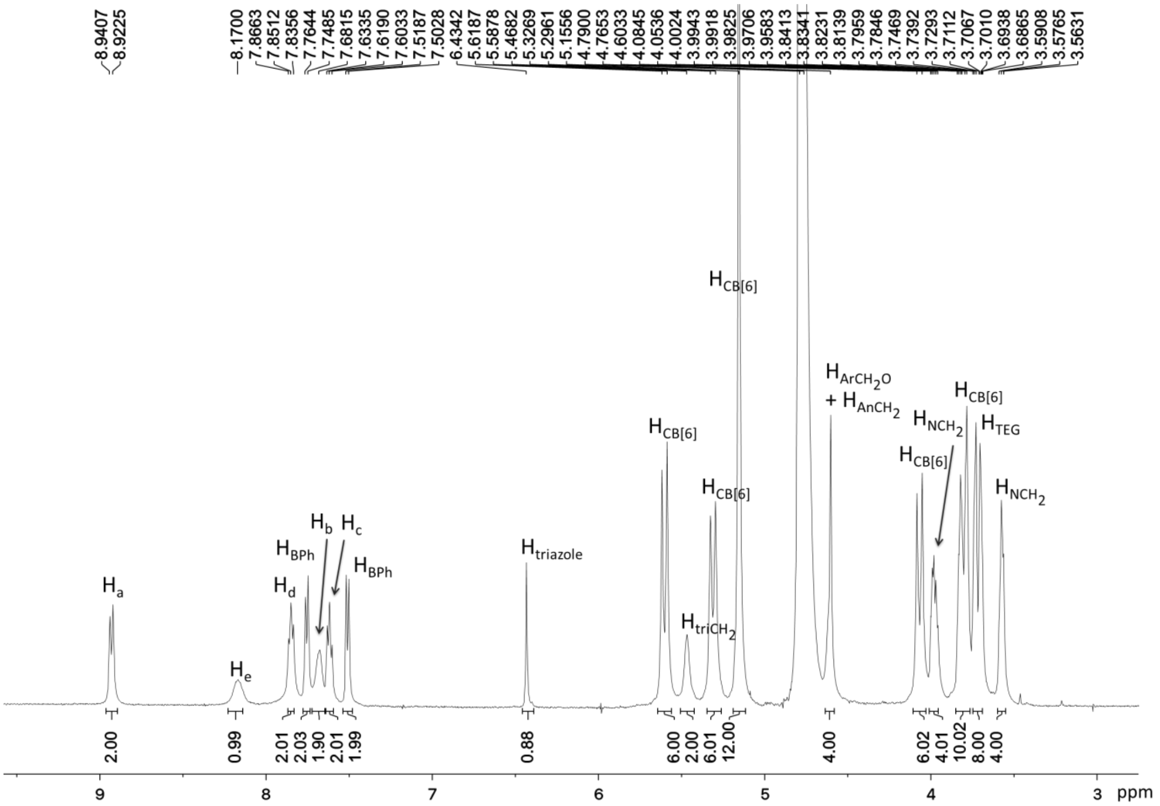

Figure S3. ^{1}H NMR (500 MHz, D_2O, 298 K) of **3R**.

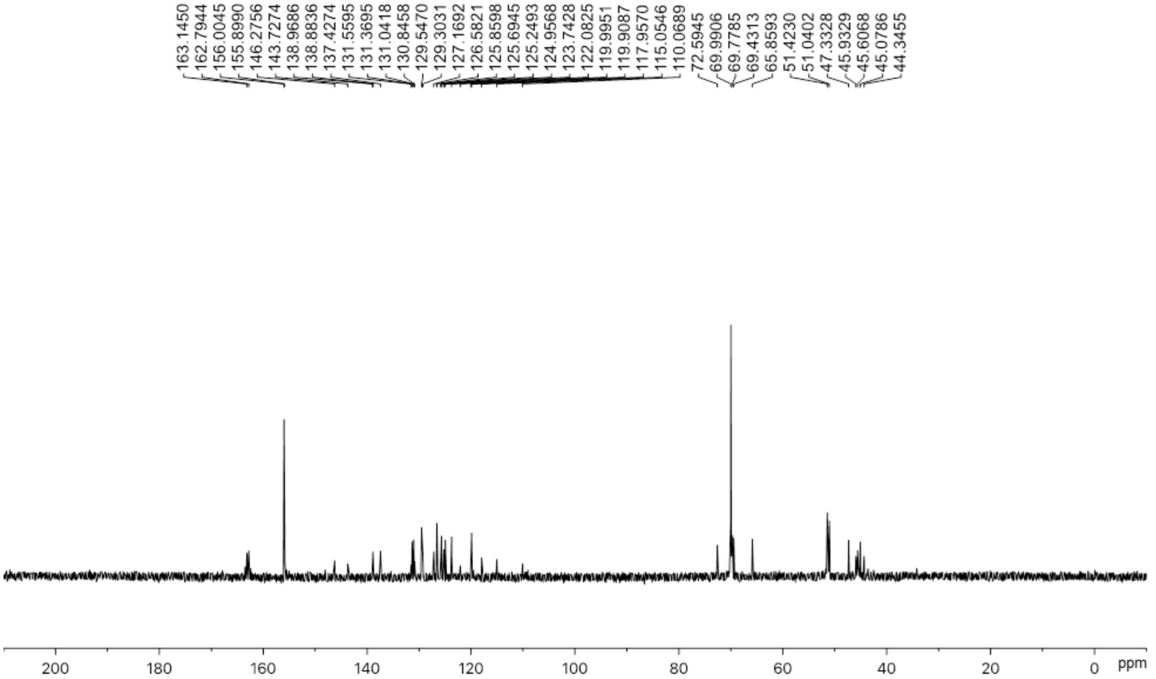

Figure S4. ^{13}C{^{1}H} NMR (100 MHz, D_2O, 298 K) of **3R**.

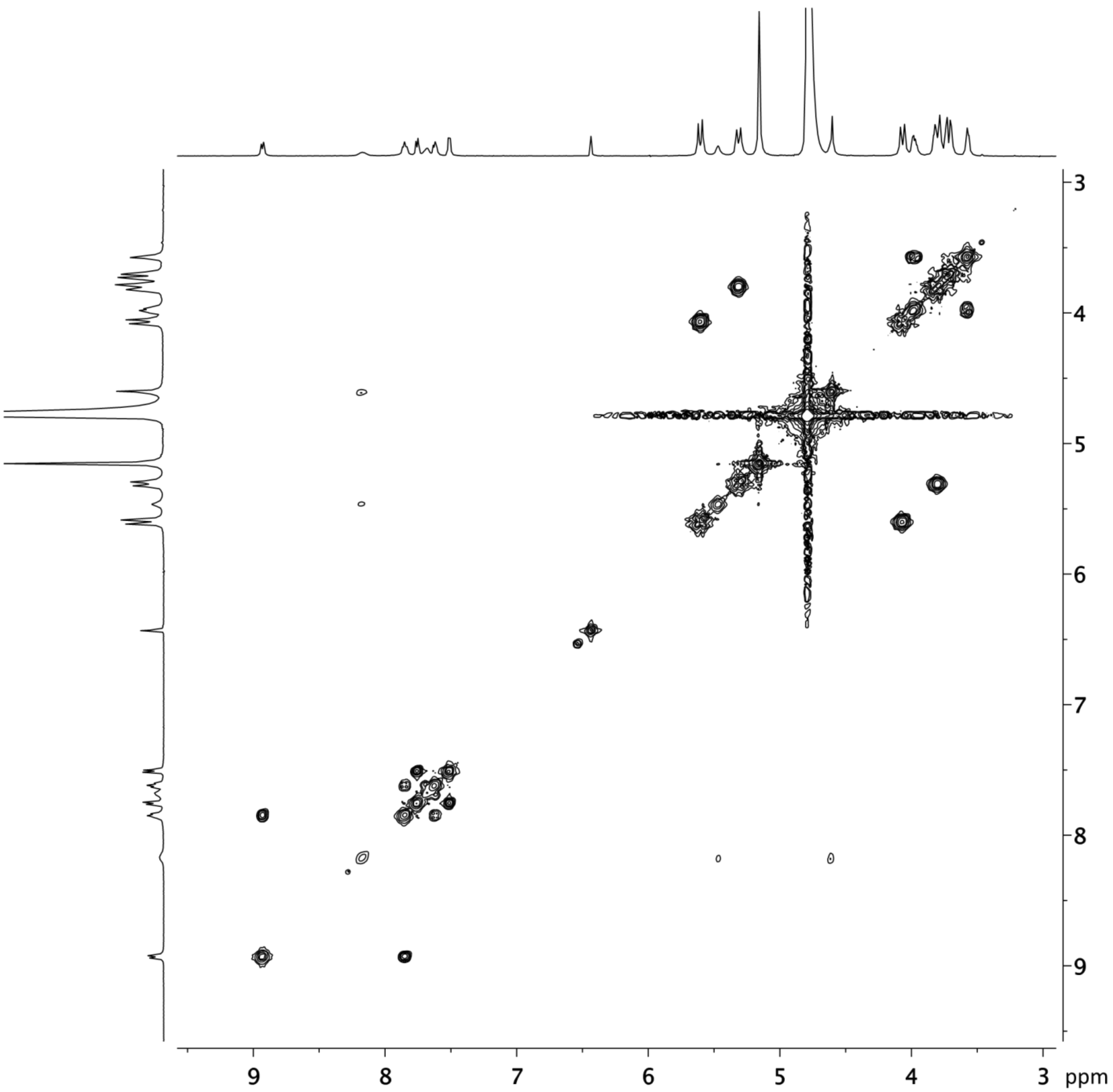

Figure S5. COSY spectrum (500 MHz, D_2O, 298 K) of **3R**.

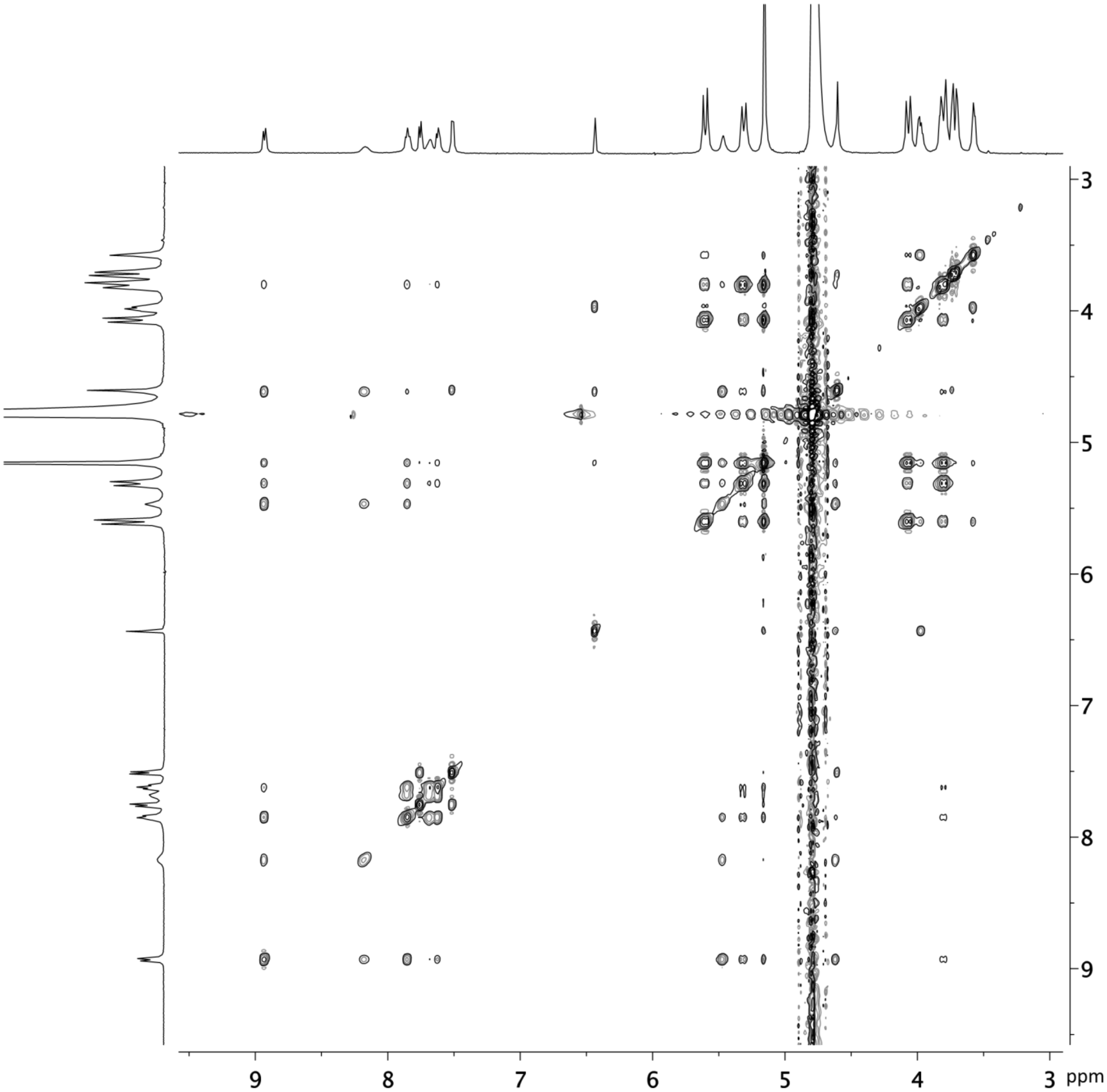

Figure S6. NOESY spectrum (500 MHz, D_2O, 298 K, mixing time: 700 ms) of **3R**.

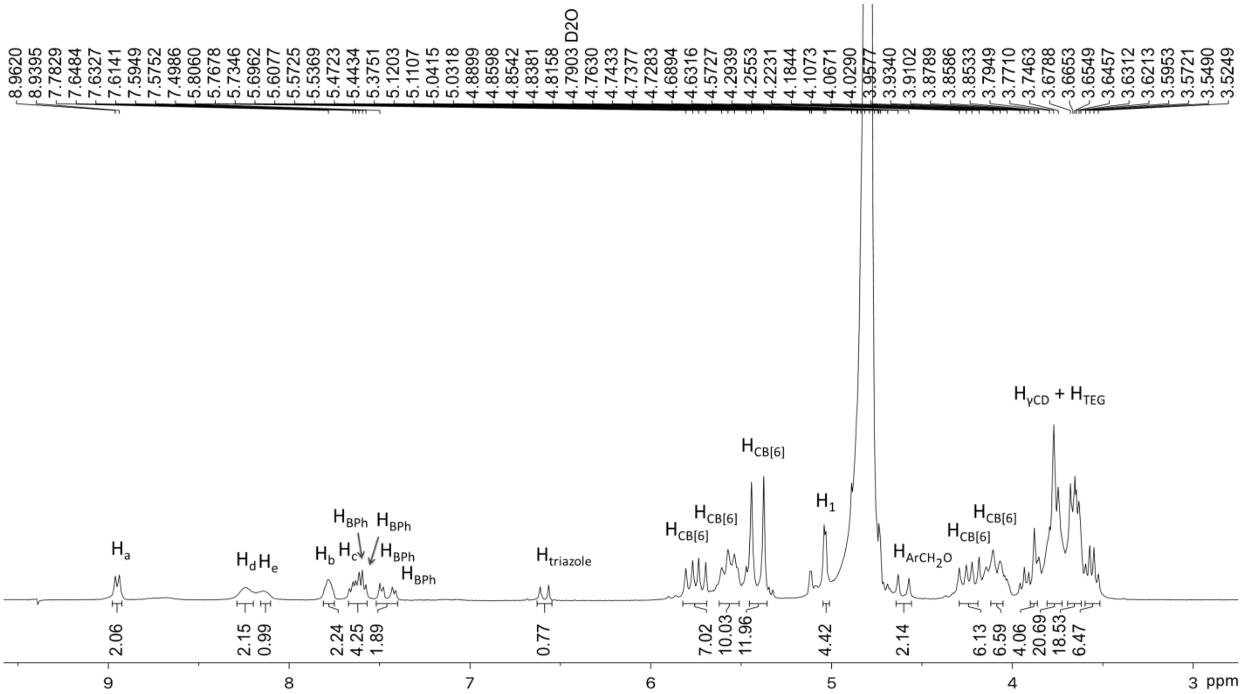

Figure S7. ^{1}H NMR (500 MHz, D_2O, 298 K) of **4R**.

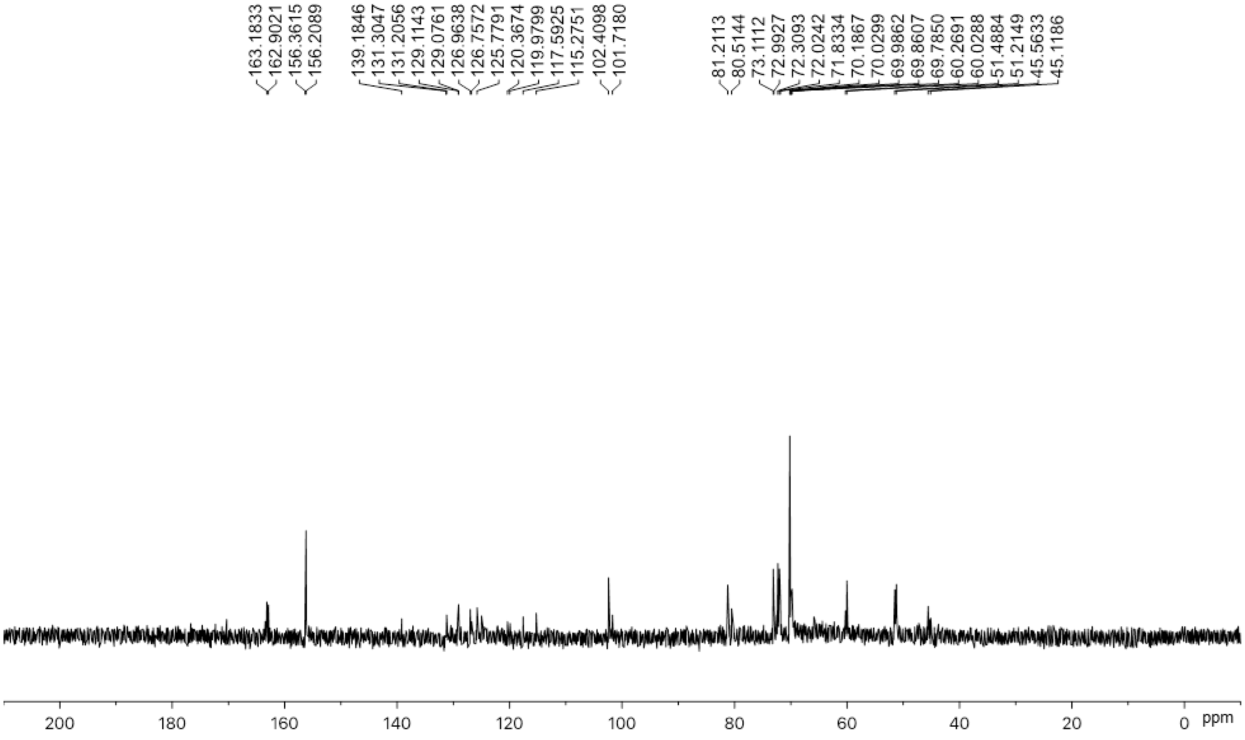

Figure S8. ^{13}C{^{1}H} NMR (125 MHz, D_2O, 298 K) of **4R**.

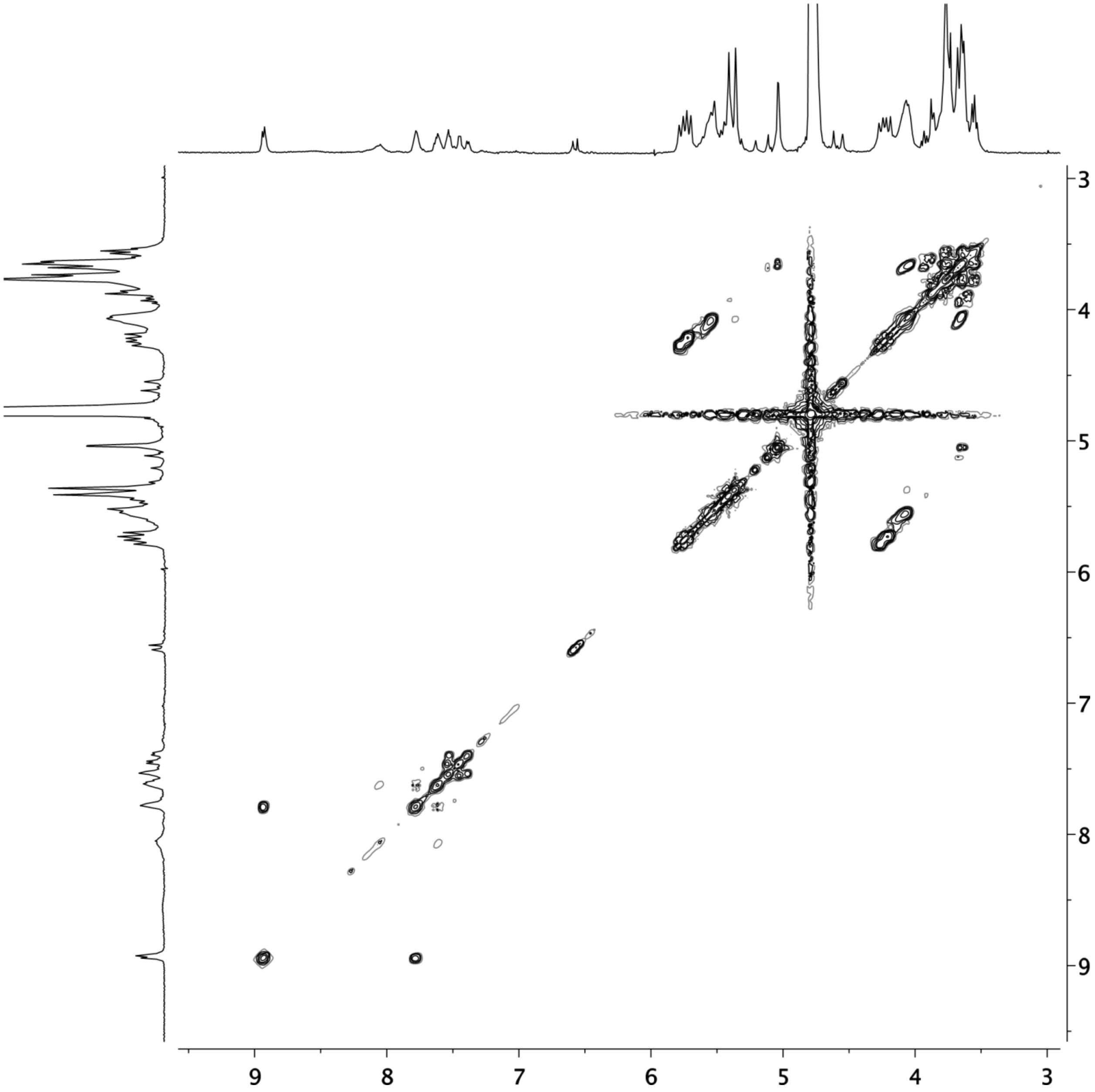

Figure S9. COSY spectrum (500 MHz, D_2O, 298 K) of **4R**.

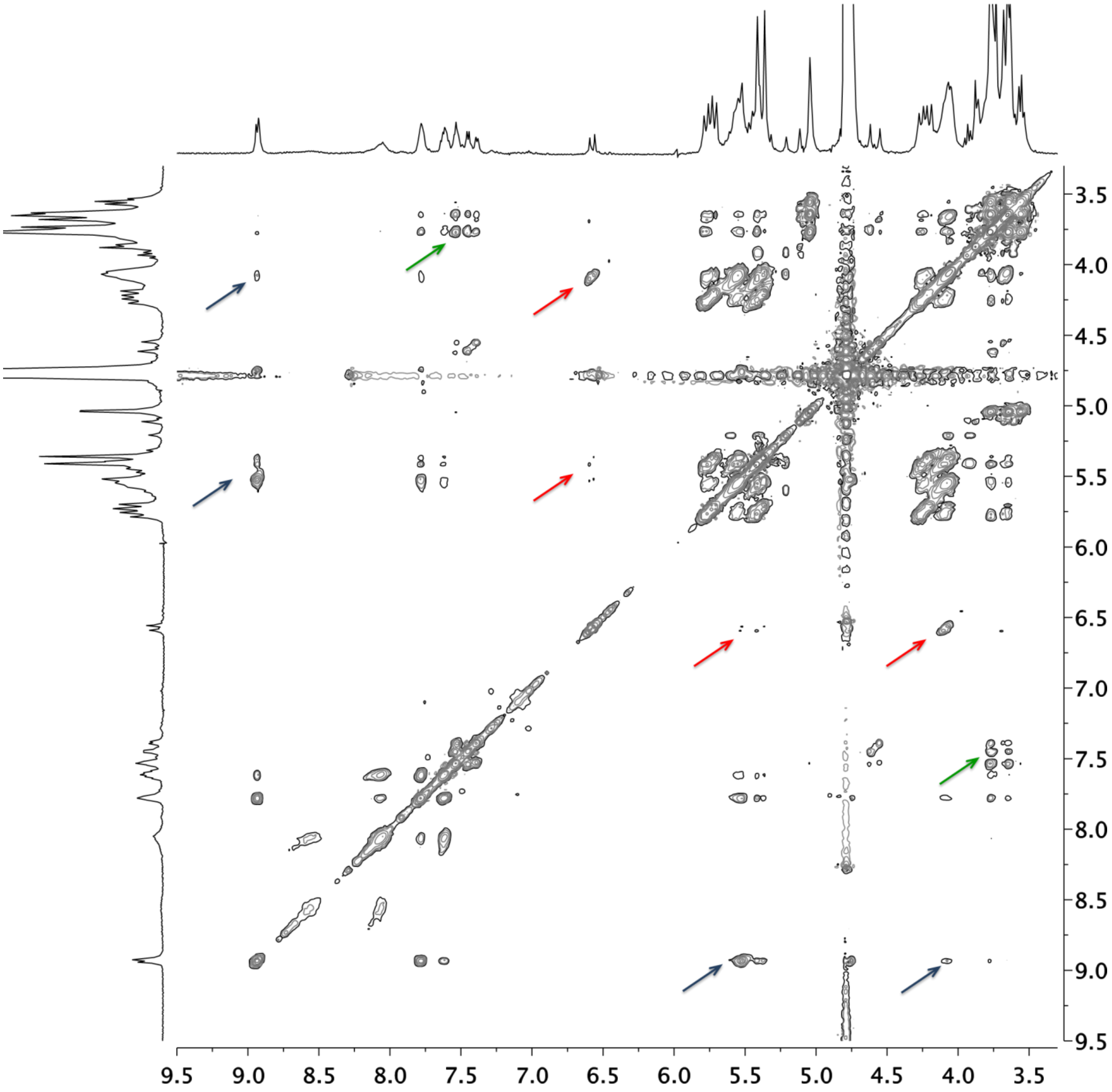

Figure S10. NOESY spectrum (500 MHz, D_2O, 298 K, Mixing time: 700 ms) of **4R**. Cross peaks between the triazole and CB[6] protons, H_a of anthracene and CB[6] protons, and biphenylene and H_2/H_3 protons are indicated by red, blue and green arrows, respectively.

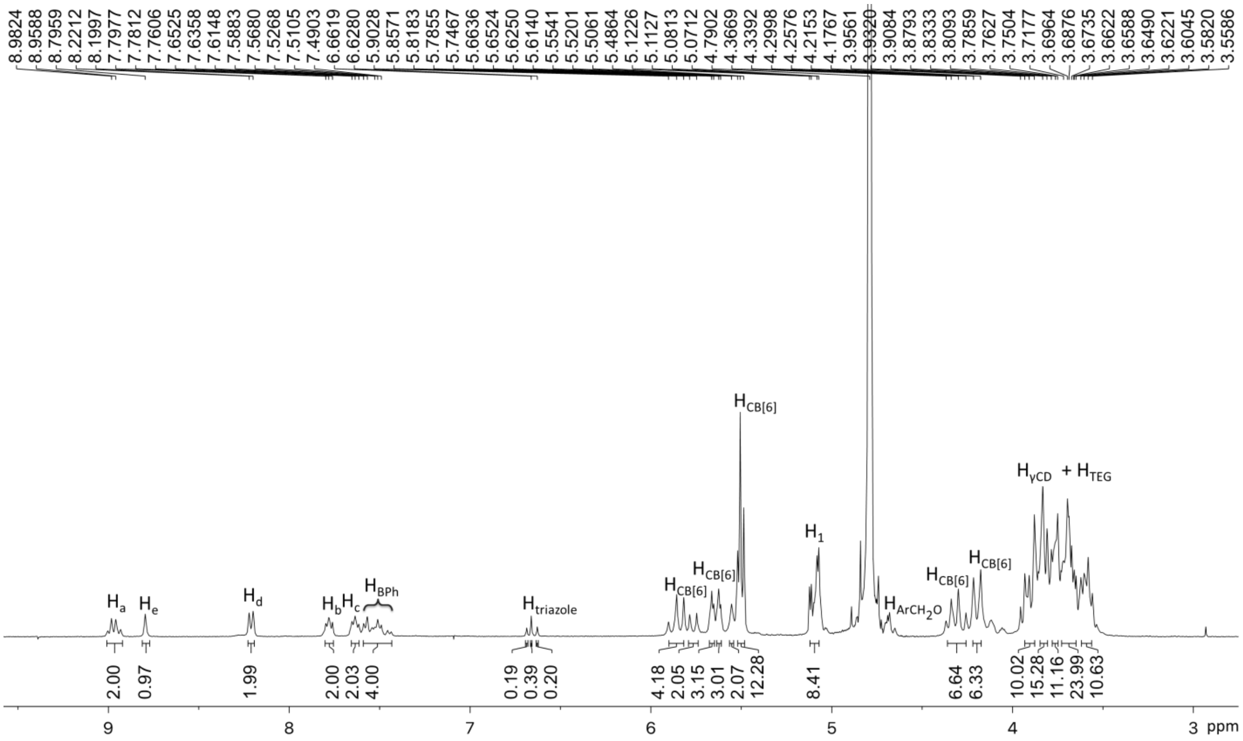

Figure S11. ^{1}H NMR (400 MHz, D_2O, 298 K) of **5R**.

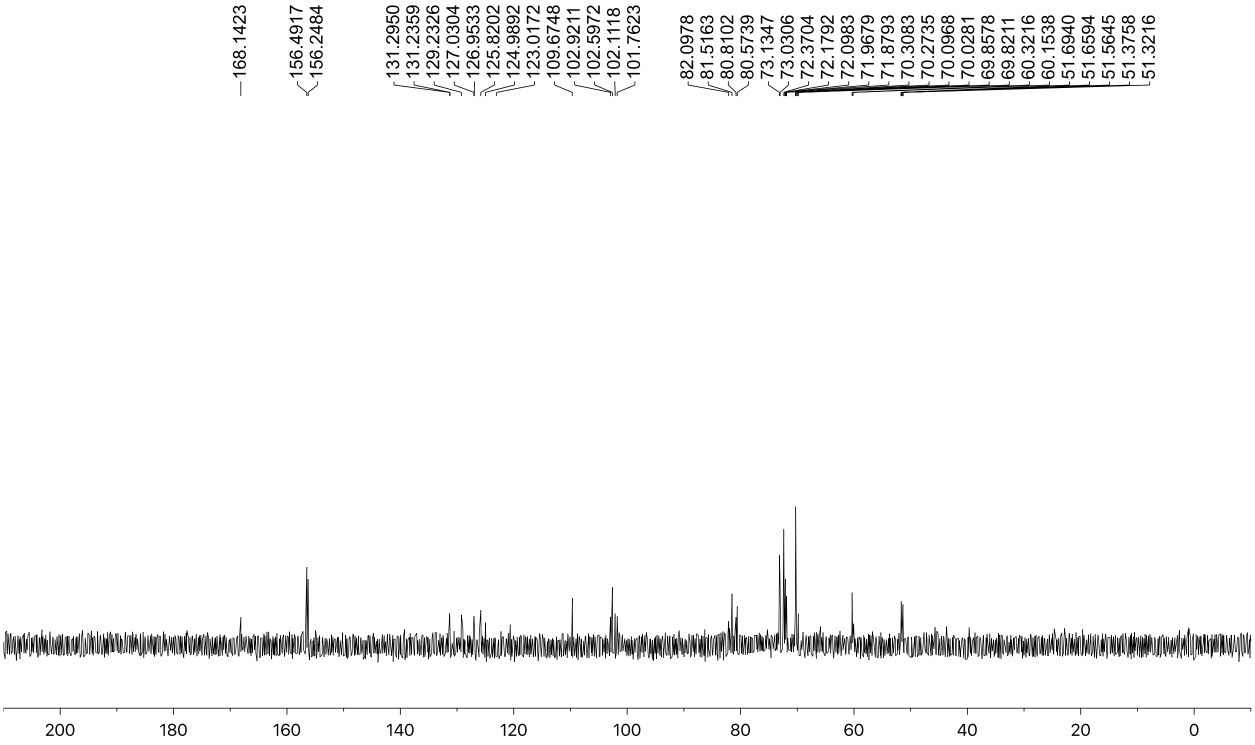

Figure S12. $^{13}C\{^{1}H\}$ NMR (125 MHz, D_2O, 298 K) of **5R**.

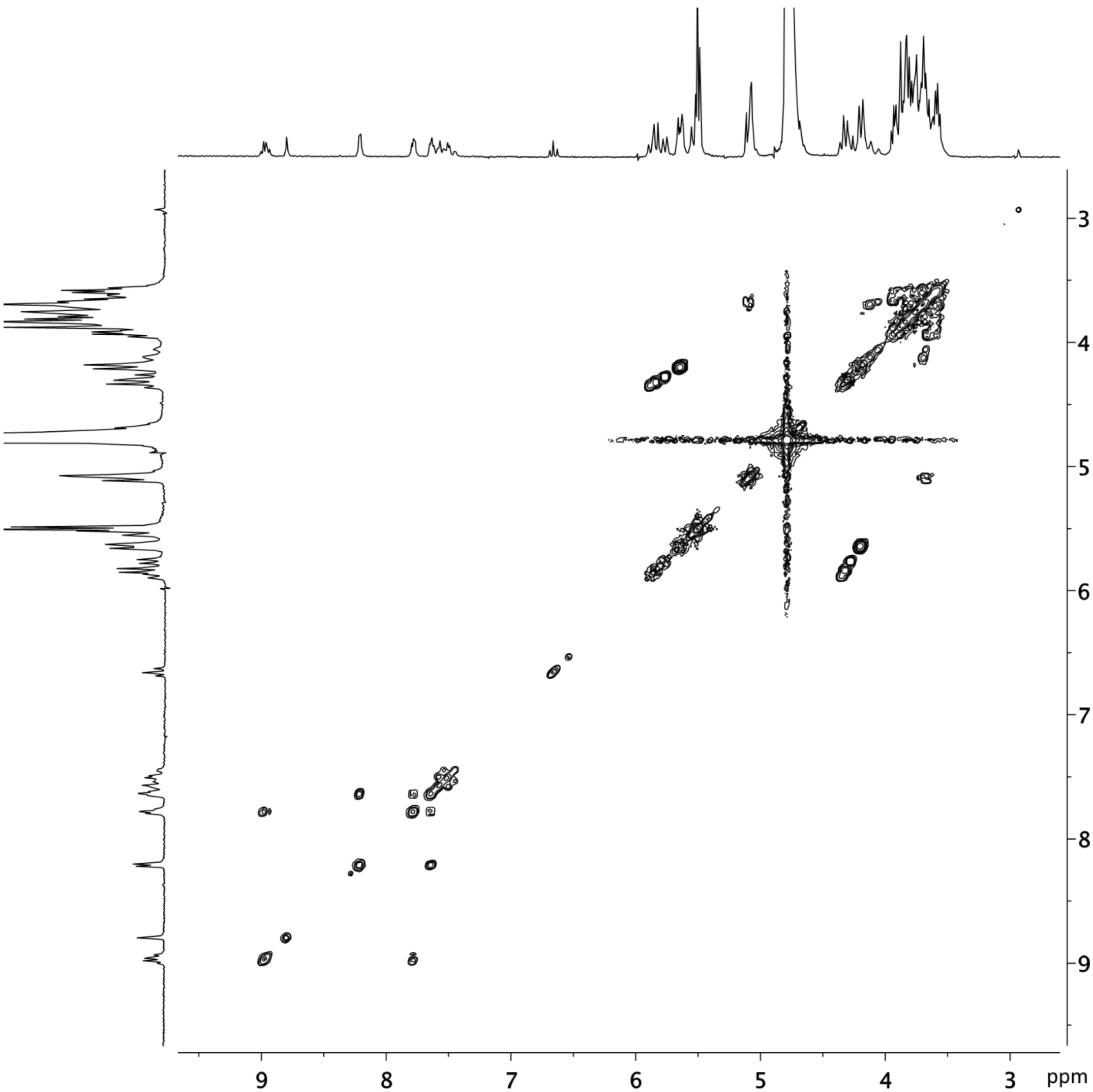

Figure S13. COSY spectrum (500 MHz, D_2O, 298 K) of **5R**.

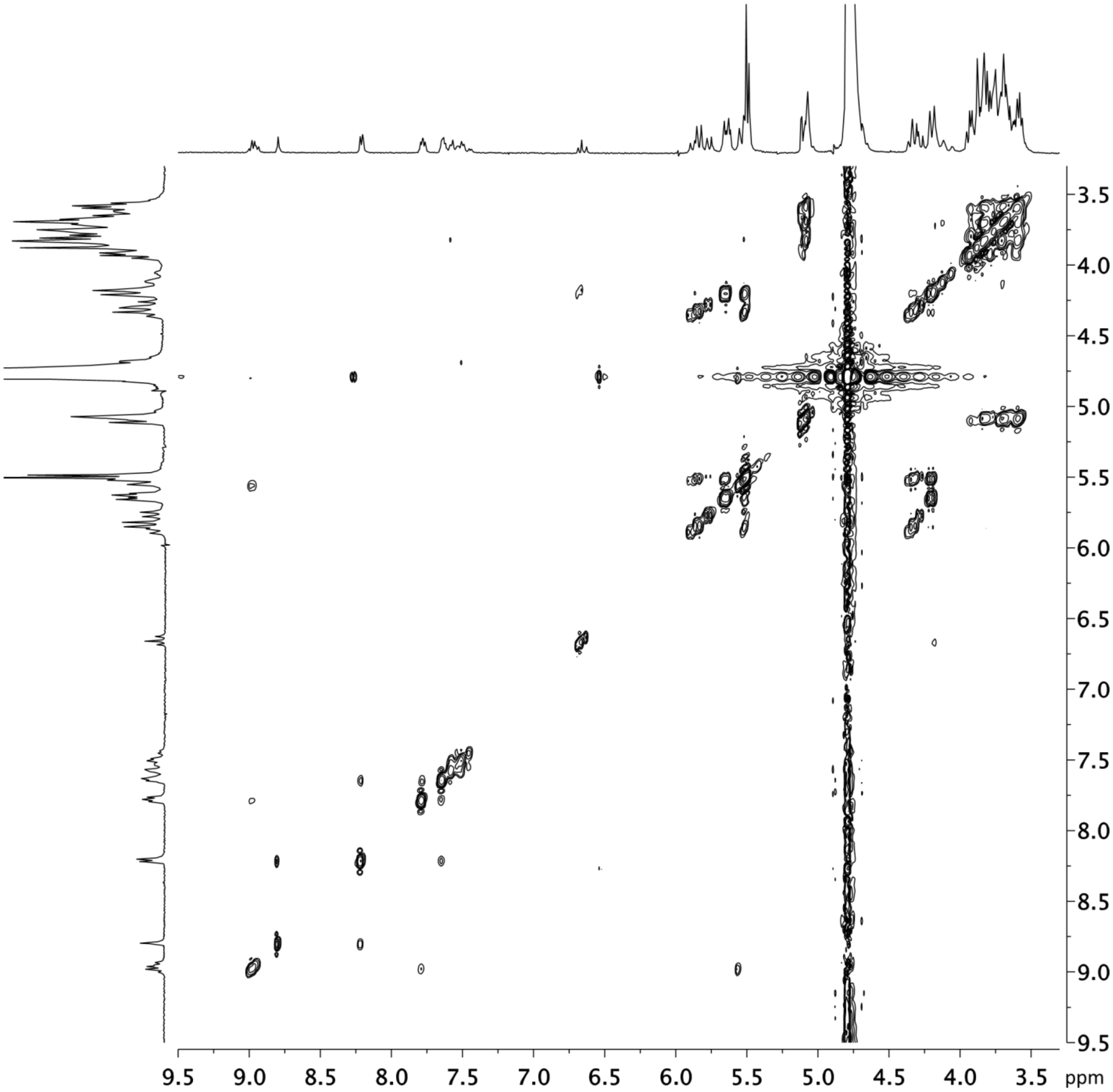

Figure S14. NOESY spectrum (500 MHz, D_2O, 298 K, mixing time: 700 ms) of **5R**.

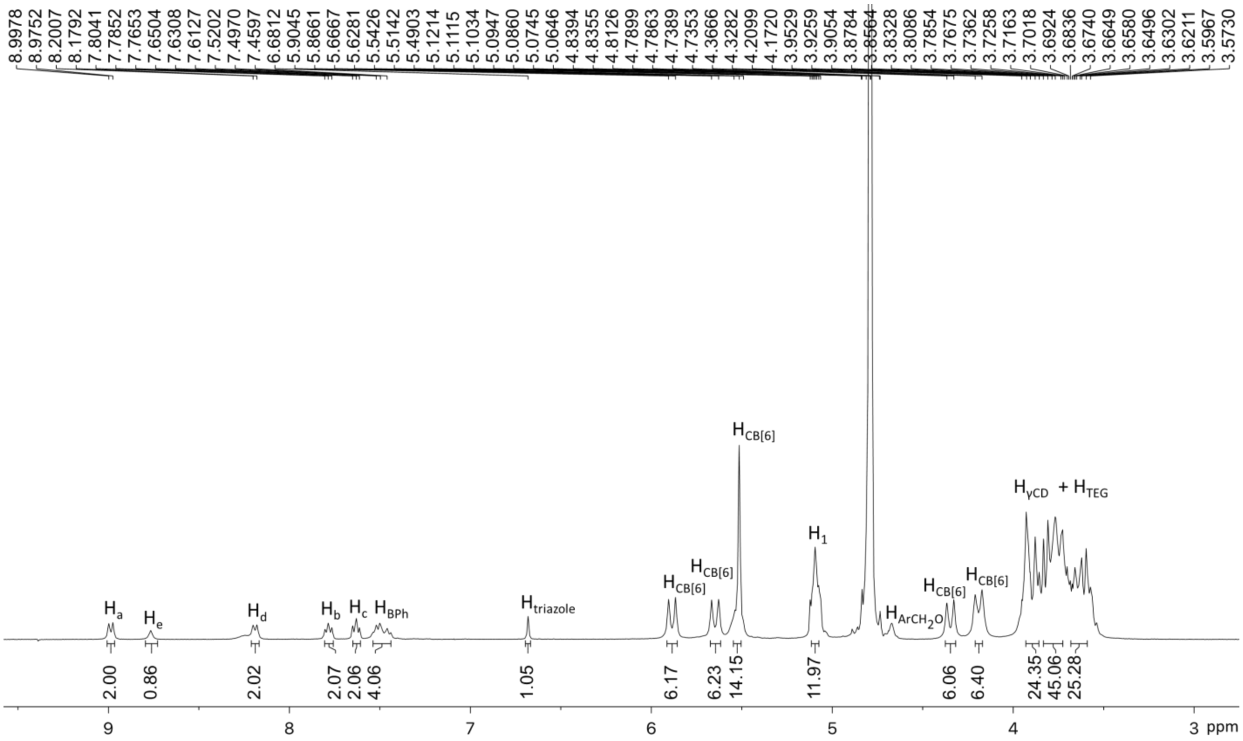

Figure S15. ^{1}H NMR (400 MHz, D_2O, 298 K) of **6R**.

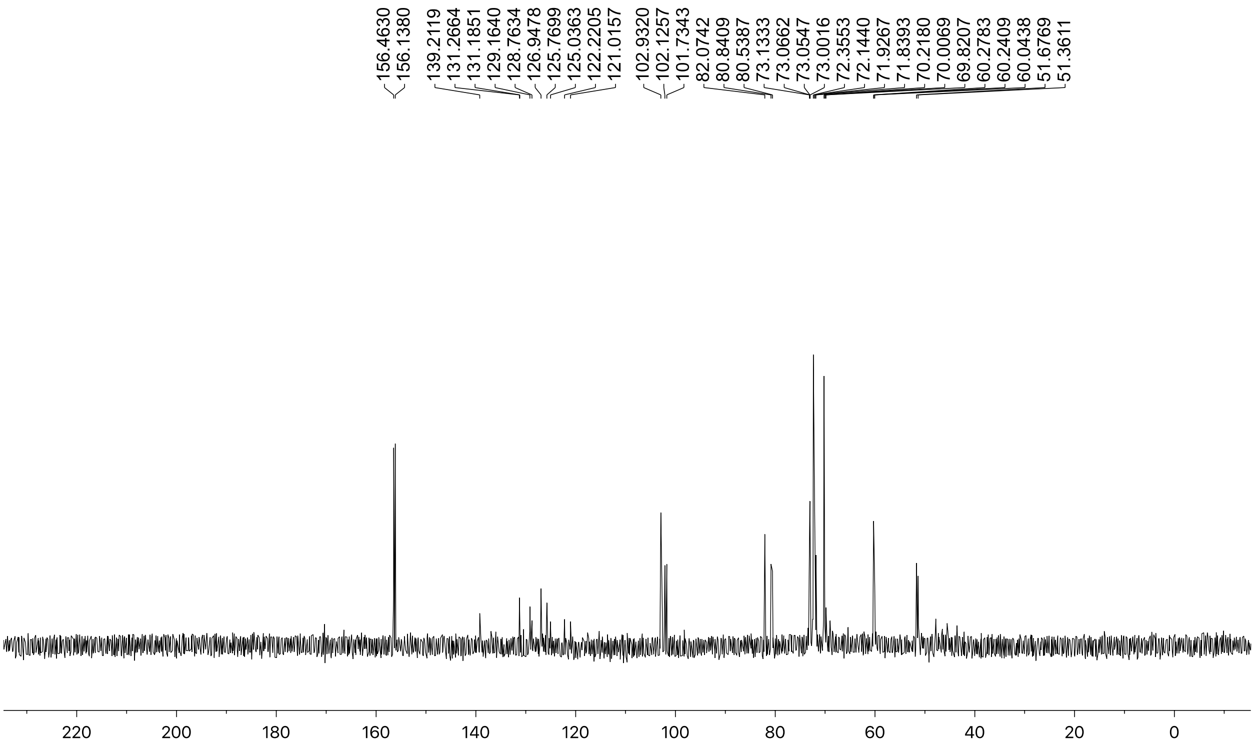

Figure S16. $^{13}C\{^{1}H\}$ NMR (125 MHz, D_2O, 298 K) of **6R**.

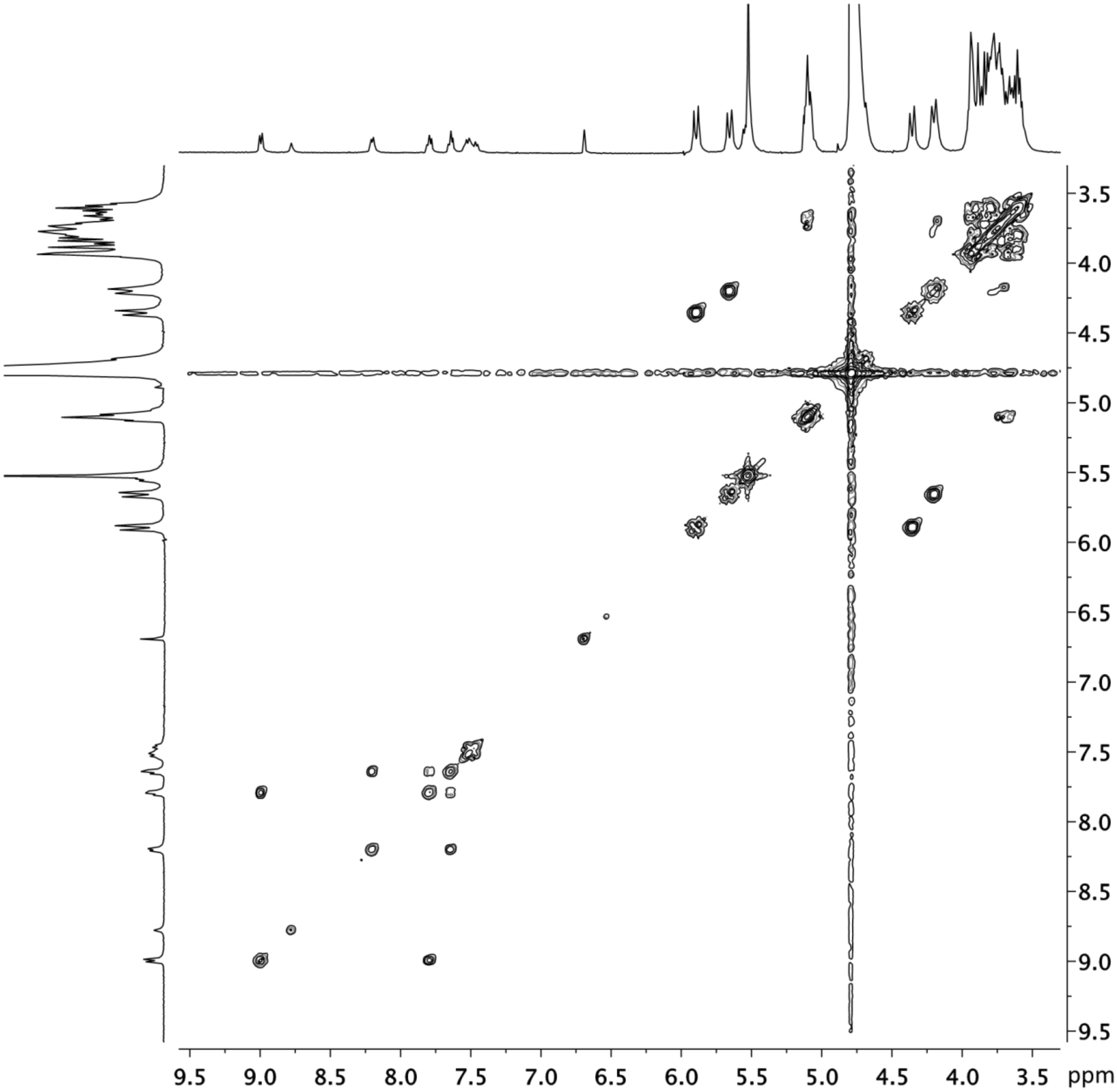

Figure S17. COSY spectrum (500 MHz, D_2O, 298 K) of **6R**.

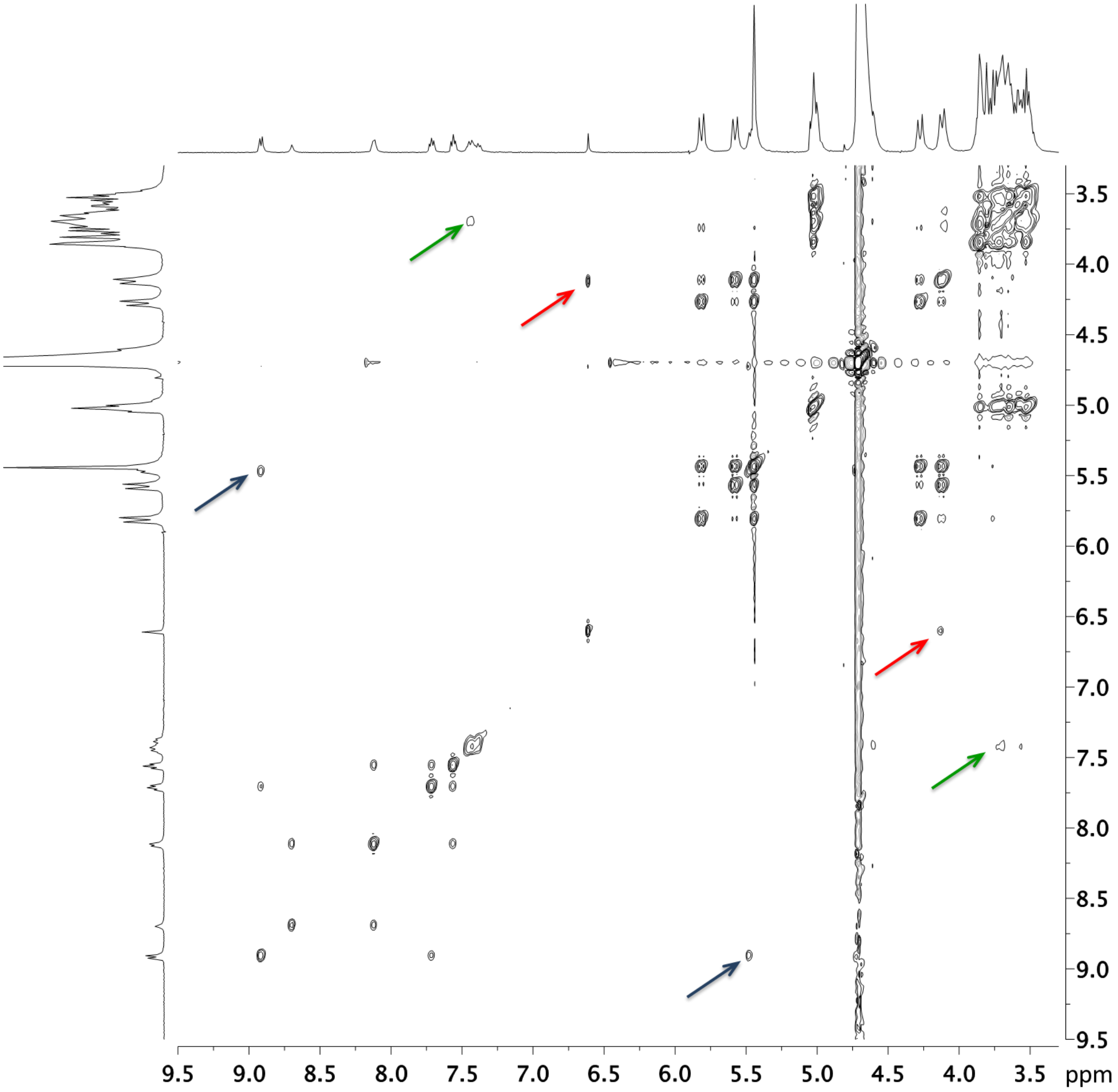

Figure S18. NOESY spectrum (500 MHz, D_2O, 298 K, mixing time: 700 ms) of **6R**. Cross peaks between the triazole and CB[6] protons, H_a of anthracene and CB[6] protons, and biphenylene and H_2/H_3 protons are indicated by red, blue and green arrows, respectively.

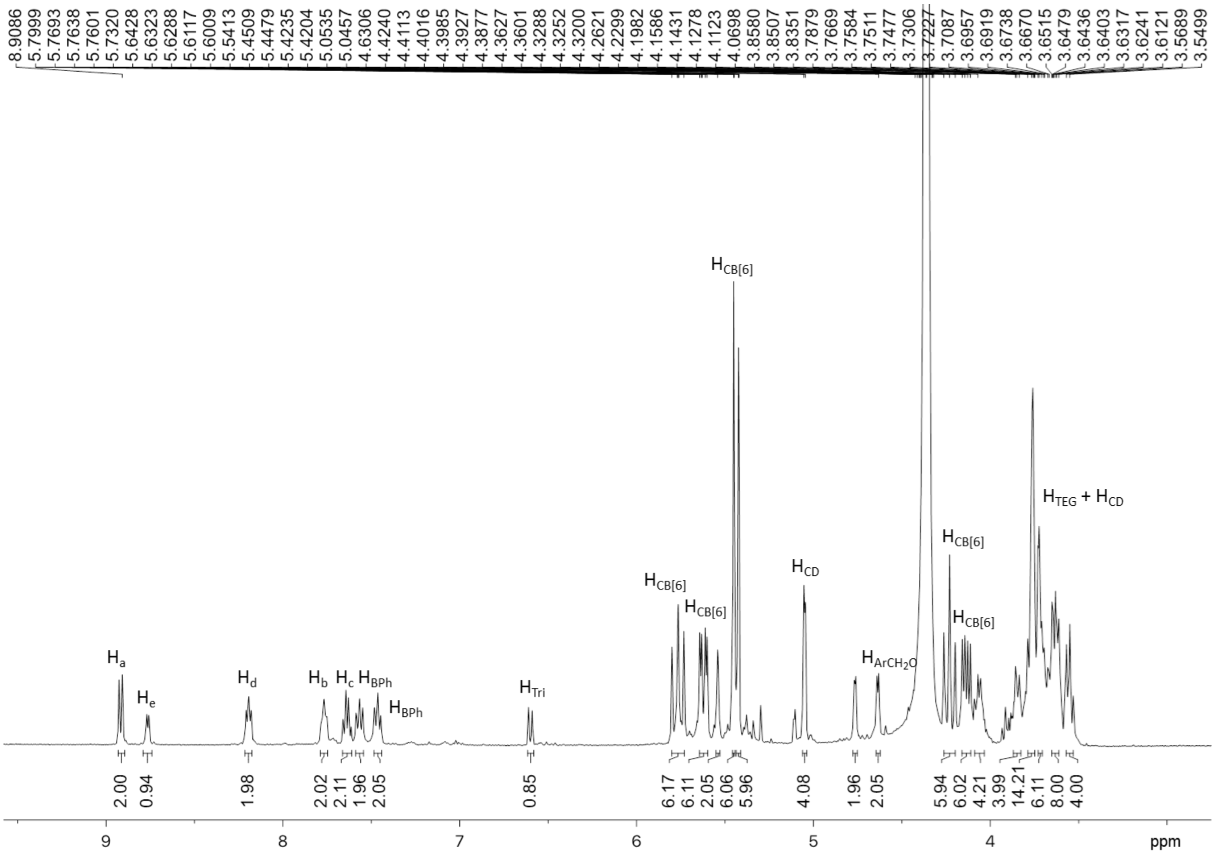

Figure S19. ^{1}H NMR (500 MHz, D_2O, 338 K) of **4R**.

3. ESIMS

Mass spectrometry was performed on a Thermo Scientific LTQ FLEET mass spectrometer or a Finnigan LCQ mass spectrometer. HRESIMS measurements were carried out on a Bruker ESI Quadrupole TOF mass spectrometer. MS^2 experiments were carried out on a Thermo Scientific LTQ FLEET mass spectrometer. Isotopic patterns were simulated using IsoPro, version 3.1.

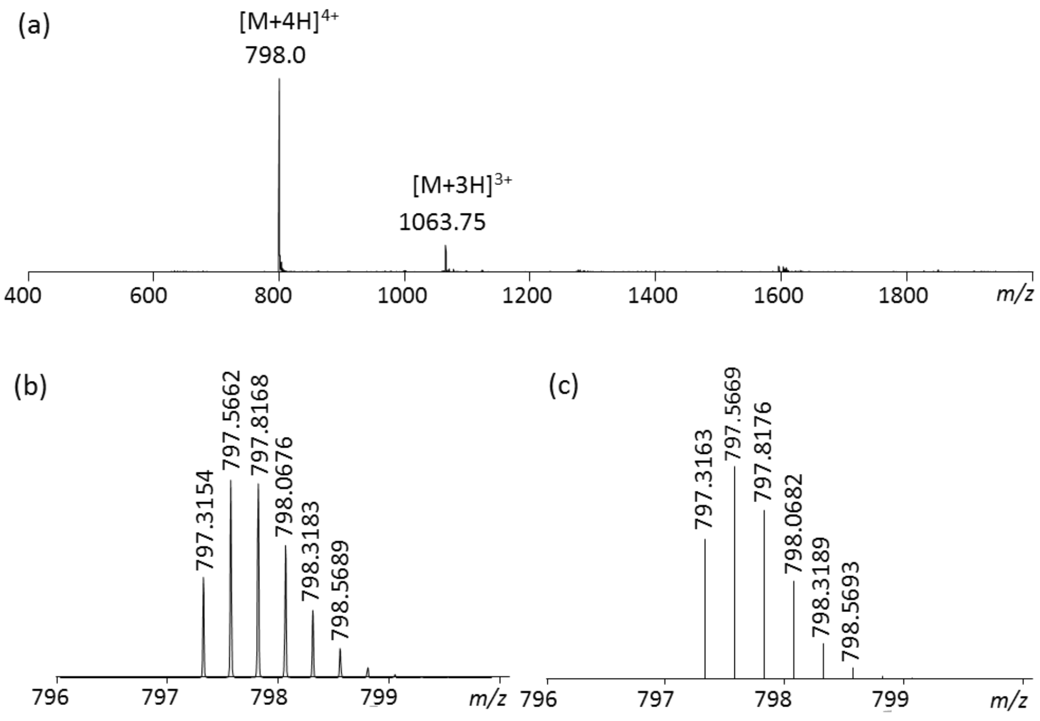

Figure S20. (a) ESIMS, (b) experimental and (c) simulated HRMS spectrum at *m/z* = 798.0 of **3R**.

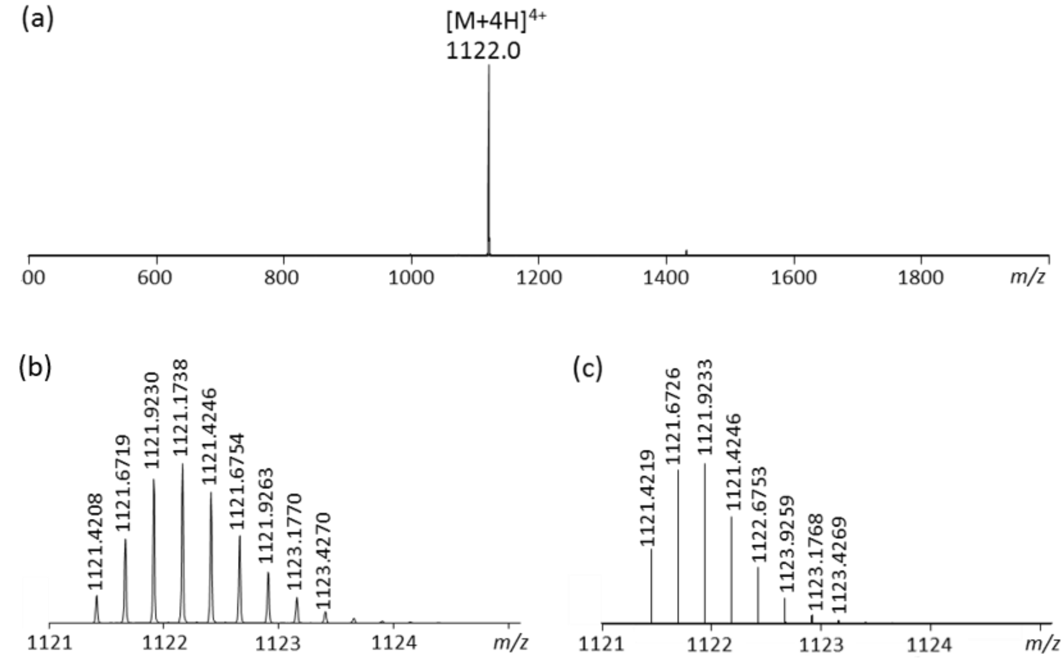

Figure S21. (a) ESIMS, (b) experimental and (c) simulated HRMS spectrum at *m/z* = 1122.0 of **4R**.

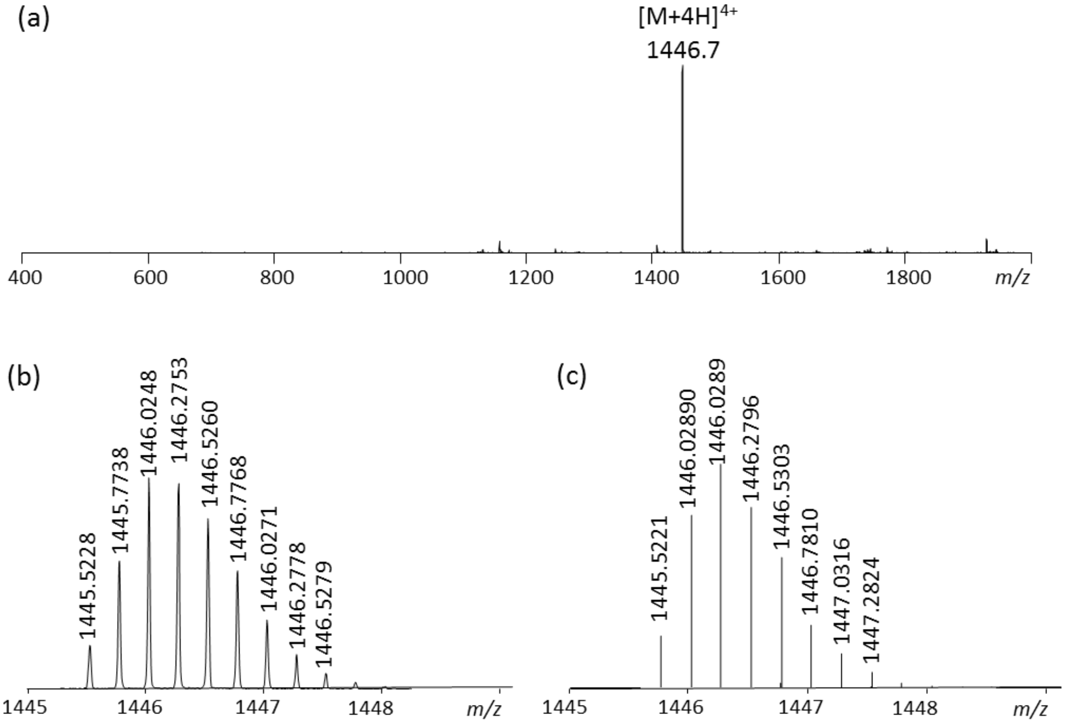

Figure S22. (a) ESIMS, (b) experimental and (c) simulated HRMS spectrum at *m/z* = 1446.7 of **5R**.

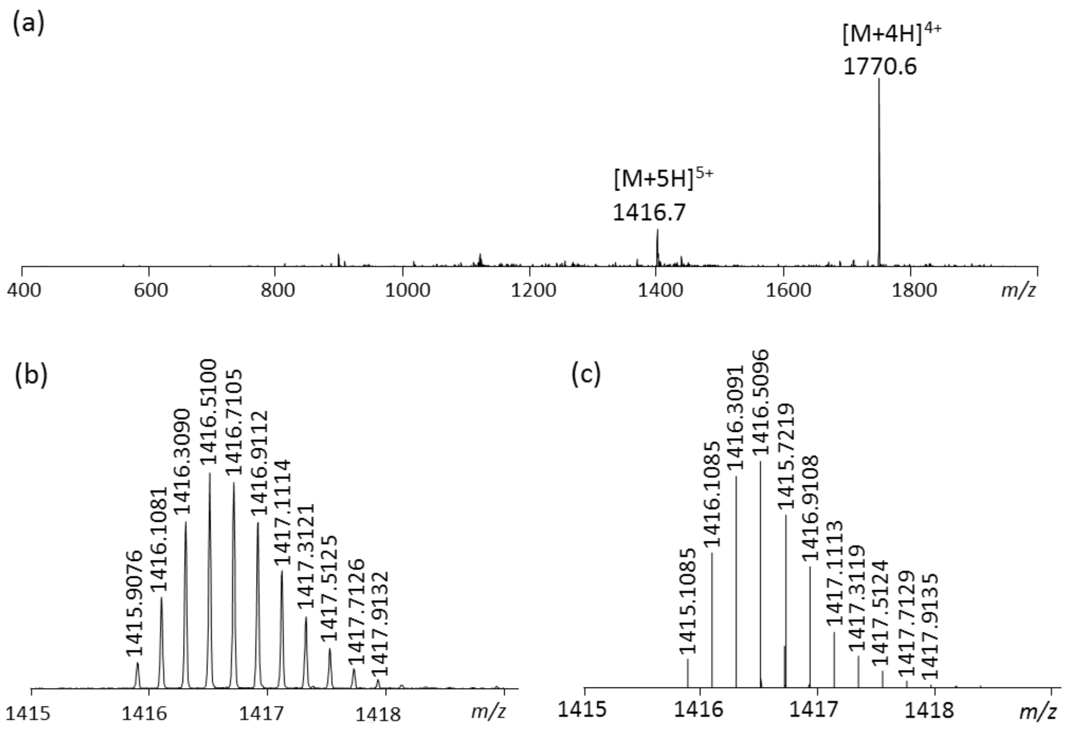

Figure S23. (a) ESIMS, (b) experimental and (c) simulated HRMS spectrum at *m/z* = 1416.7 of **6R**.

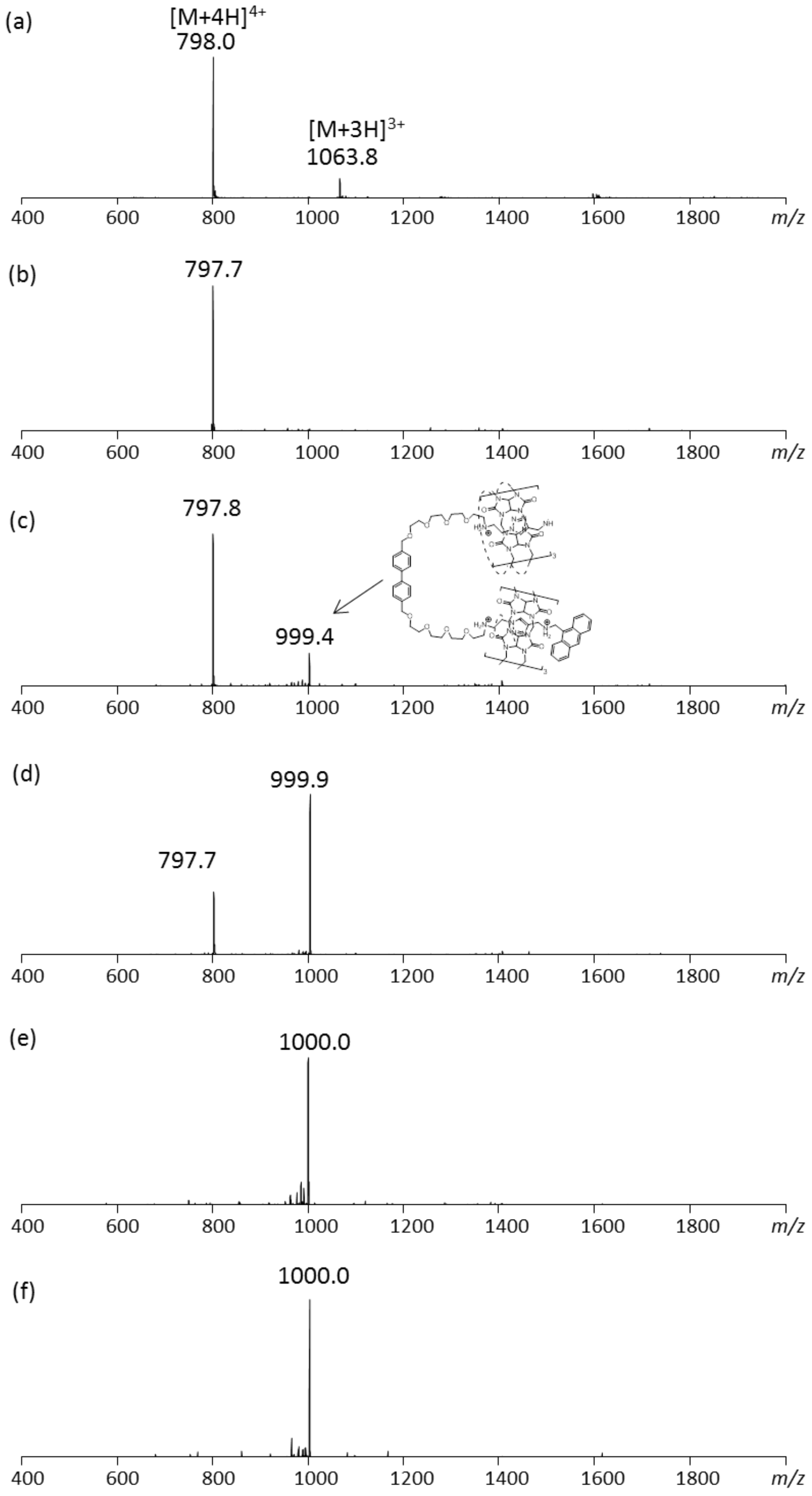

Figure S24. (a) Parent ESIMS spectrum; and MS^2 spectra (for the peak at m/z = 798.0) at normalized collision energy of (b) 17%, (c) 19%, (d) 21%, (e) 23% and (f) 25% of **3R**.

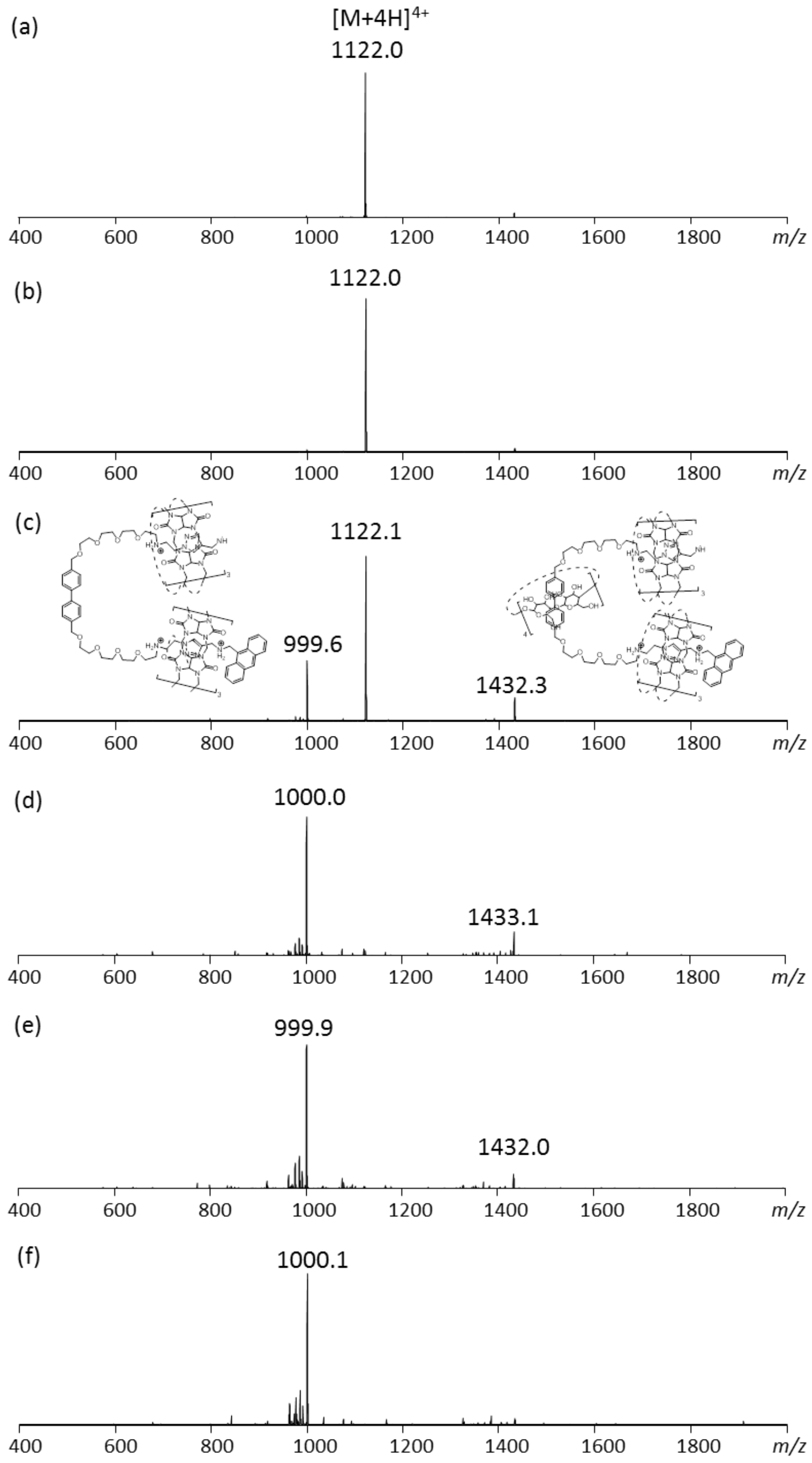

Figure S25. (a) ESIMS spectrum; and MS^2 spectra (for the peak at m/z = 1122.0) of **4R** at an normalized collision energy of (b) 17%, (c) 19%, (d) 21%, (e) 23% and (f) 25%.

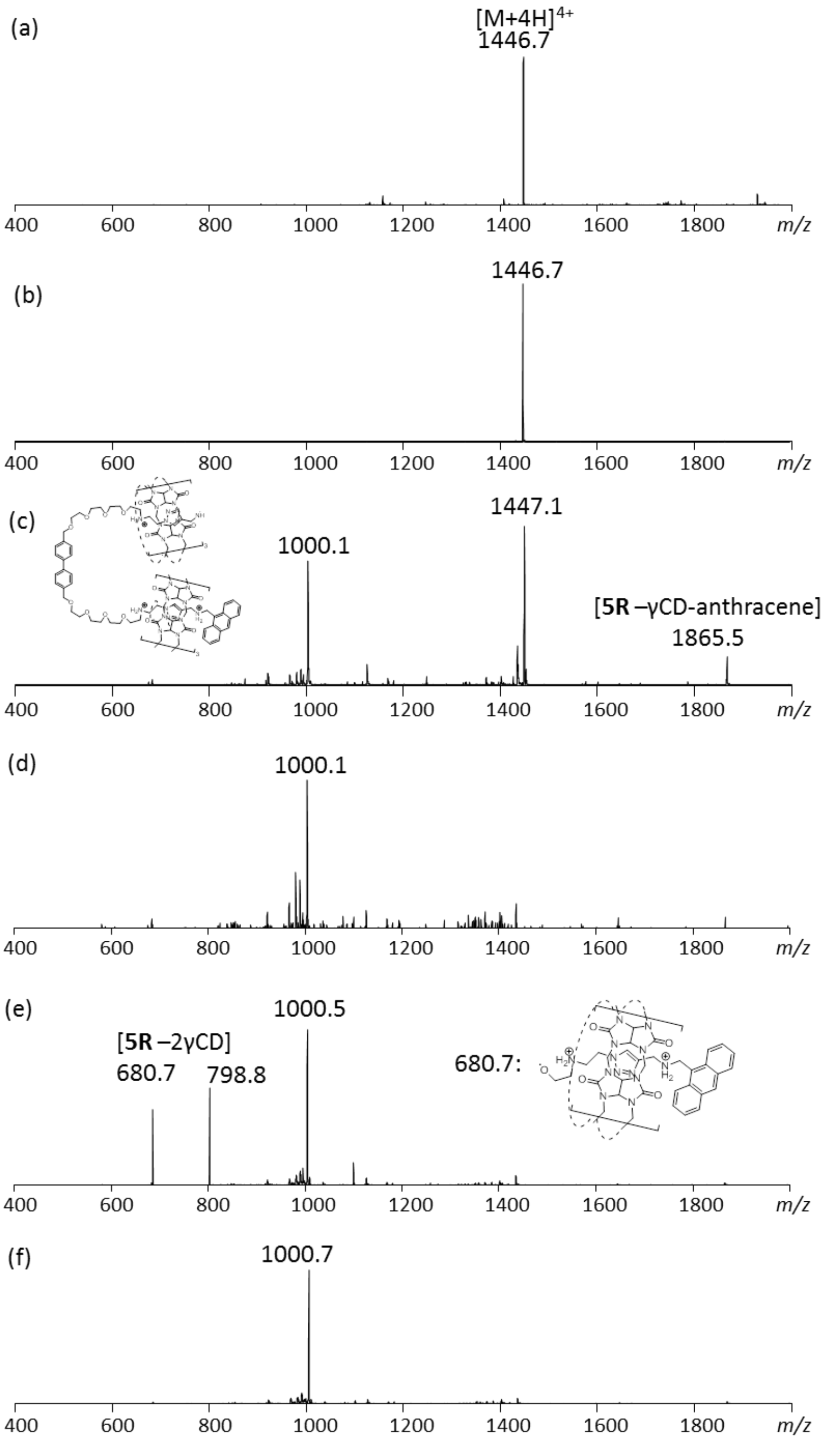

Figure S26. (a) ESIMS spectrum; and MS^2 spectra (for the peak at *m/z* = 1446.7) of **5R** at an normalized collision energy of (b) 17%, (c) 19%, (d) 21%, (e) 23% and (f) 25%.

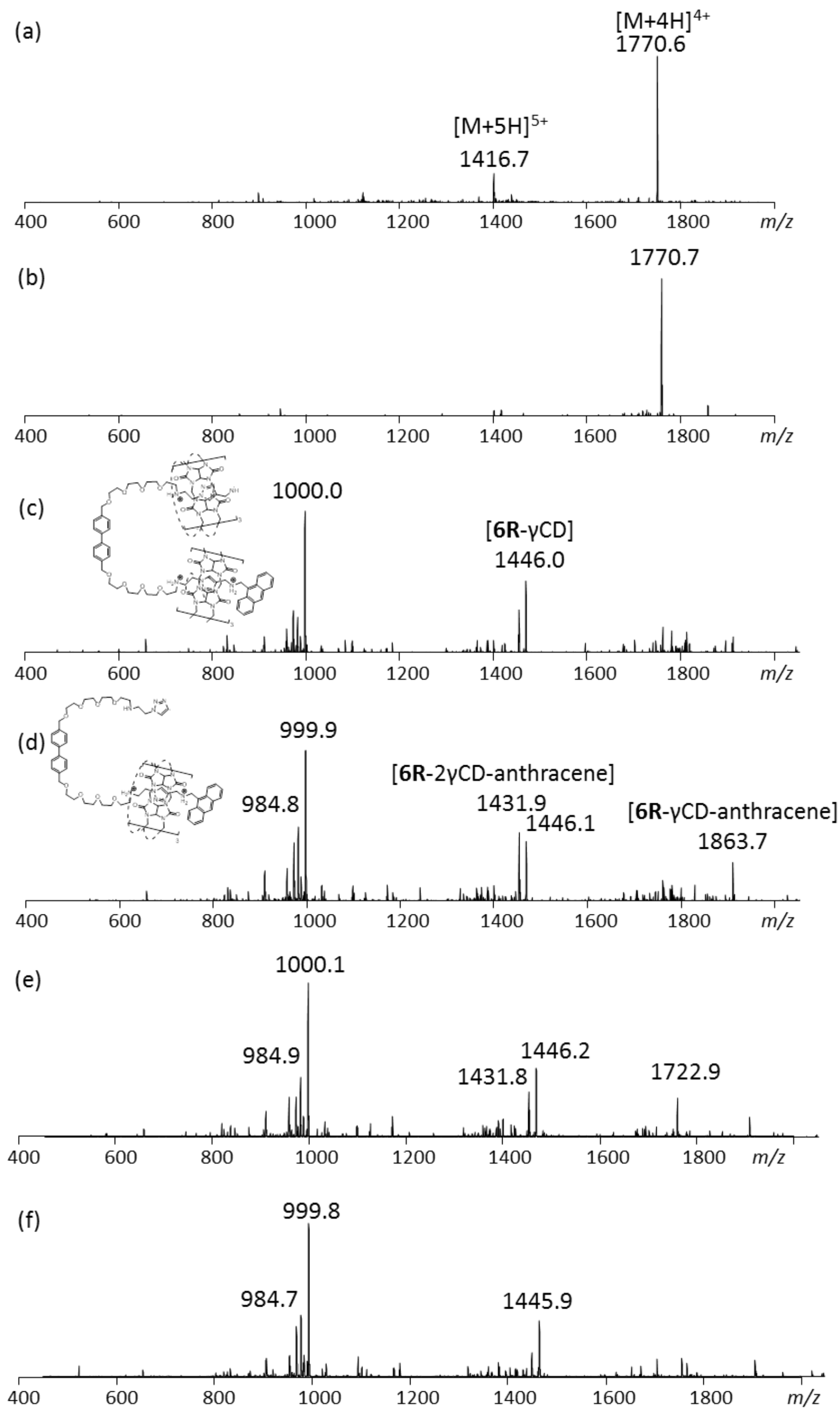

Figure S27. (a) ESIMS spectrum; and MS^2 spectra (for the peak at *m/z* = 1446.7) of **6R** at an normalized collision energy of (b) 17%, (c) 19%, (d) 21%, (e) 23% and (f) 25%.

4. References

1. Ng, A. W. H.; Yee, C.-C.; Wang, K.; Au-Yeung, H. Y. *Beilstein J. Org. Chem.* **2018**, *14*, 1846-1853
2. Angelos, S.; Yang, Y.-W.; Patel, K.; Stoddart J. F.; Zink, J. I. *Angew. Chem. Int. Ed.* **2008***, 47,* 2222−2226.
